# Supplementary material for: NIR-dye bridged human serum albumin reassemblies for effective photothermal therapy of tumor
Source: Nat Commun. 2023 Oct 17;14:6567. doi: 10.1038/s41467-023-42399-9 (PMC10582160; doi:10.1038/s41467-023-42399-9)
Supplement: Supplementary file 1 — Supplementary Information [file 41467_2023_42399_MOESM1_ESM.pdf]

Supplementary Information

NIR-dye bridged human serum albumin reassemblies for effective photothermal therapy of tumor

Shi *et al.*

Section S1: Materials and Methods

Materials

Ultra-pure water was obtained from Merck Milli-Q IQ 70XX systems (Germany). Human serum albumin lyophilized powder (≥ 96%) and tris(2-carboxyethyl) phosphine hydrochloride (TCEP) were purchased from Sigma-Aldrich (USA). Dimethyl sulfoxide (DMSO, ≥ 99.5%) was purchased from Solarbio (China). Dichloromethane (DCM) was refluxed with CaH_2 overnight before use. Ethanol was refluxed with magnesium and iodine overnight and distilled before use. Ammonium acetate, 1-(4-(3-azidopropoxy)phenyl)ethan-1-one, $BF_3 \cdot Et_2O$, butanol, 9-julolidinecarboxaldehyde, nitromethane, and triethylamine (TEA) were obtained from Sigma Aldrich and used without further purifications. DBCO-Maleimide was obtained from Click Chemistry Tools. Sulfo-Cy5-mal was provided by Xi'an Ruixi Biological Technology (China). DMEM-H medium, RPMI-1640 medium, PBS and 0.25% trypsin solution (with EDTA) were obtained from Gibco, Thermo Fisher Scientific (USA). FBS was purchased from NEWZERUM (New Zealand). Annexin-V/PI apoptosis kit, calcein-AM/PI live dead cell staining kit, and Hoechst 33342 staining solution were obtained from Beyotime (China). mβ-CD, Genistein, Dynasore, Chloroquine, Chlorpromazine, Amiloride, and Simvastatin were purchased from Aladdin (China).

Characterizations

All chemical structures were confirmed by ^{1}H and ^{13}C NMR spectra and high-resolution mass spectra (HRMS) spectrometry. ^{1}H and ^{13}C nuclear magnetic resonance (NMR) spectra were measured on a Bruker BBFO 400/600 spectrometer using deuterated chloroform ($CDCl_3$) as the solvents. High-resolution mass spectrometry (HRMS) was performed on a Q-tof Premier MS spectrometer (Waters) and a MAT95XP mass spectrometer (Thermo Fisher Scientific). The *in vivo* and *ex vivo* living images were obtained by IVIS Lumina II (PerkinElmer). The photothermal conversion efficiency (PCE) was determined by analyzing the heating and cooling curves according to the following equations[1].

$$\eta = \frac{hs\Delta Tmax - Qs}{I(1-10^{-A808})} \quad (1)$$

$$hs = \frac{mC}{\tau} \quad (2)$$

$$t = -\tau ln\,(\theta) \quad (3)$$

$$\theta = \frac{\Delta T}{\Delta Tmax} \quad (4)$$

where η is PCE, *h* represents heat transfer coefficient, *s* represents surface area, *Qs* represents the heat-related to the light absorbance of the deionized water, *I* represents the power of the laser, *A808* represents the absorbance of sample in 808 nm. The *hs* could be calculated by equation (2), where m represents the mass of the sample, *C* represents the heat capacity of water, and τ could be calculated by the cooling curves according to equation (3) and (4).

Synthesis

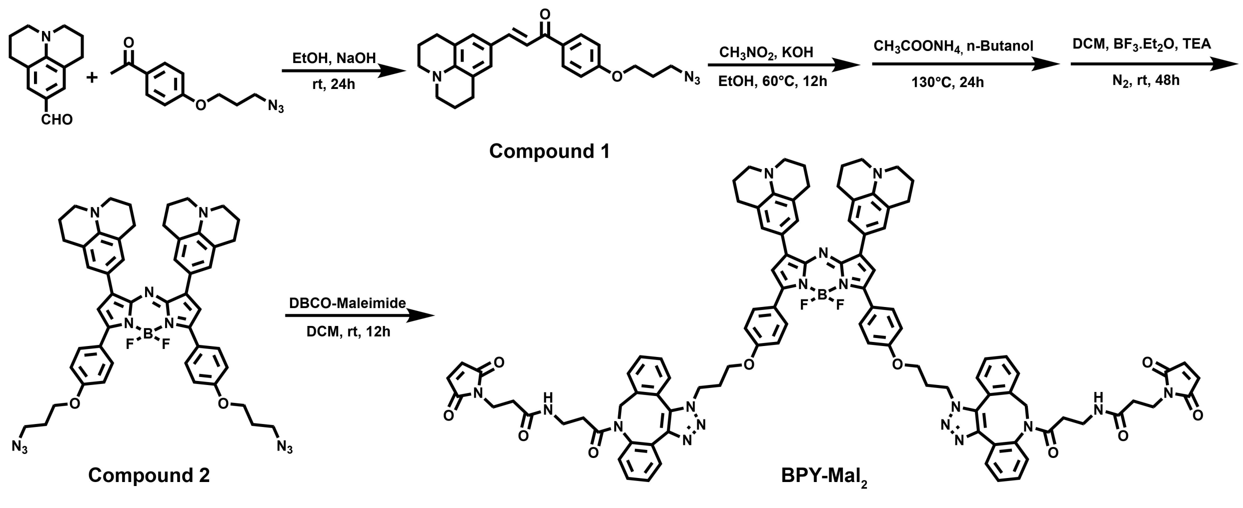

Supplementary Fig. 1. Synthetic route of BPY-Mal_2.

Compound 1

9-Julolidinecarboxaldehyde (1 eq, 1.3 g, 6.46 mmol) and 1-(4-(3-azidopropoxy)phenyl)ethan-1-one (1 eq, 1.42 g, 6.46 mmol) were dissolved in absolute ethanol (10 mL). Aqueous sodium hydroxide solution (3 eq, 0.76 g, 19.38 mmol, 10 mL) was added. Resulting mixture was stirred at room temperature for 24 h, during which the product precipitated. The solution was poured into 1 M HCl (10 mL), and further acidified with concentrated HCl. The crude product was washed with water to obtain a white solid used for further synthesis without purification (2.2 g, 84.6%). ^{1}H NMR (400 MHz, $CDCl_3$): δ 8.02 (s, 2H), 7.72 (s, 1H), 7.29 (s, 1H), 7.11 (s, 2H), 6.96 (s, 2H), 4.13 (s, 2H), 3.54 (s, 2H), 3.24 (s, 4H), 2.76 (s, 4H), 2.09 (s, 2H), 1.97 (s, 4H). ^{13}C NMR (100 MHz, $CDCl_3$): δ 188.20, 163.44, 146.06, 144.65, 132.78, 130.50, 128.06, 121.79, 121.01, 115.50, 114.07, 65.60, 51.76, 48.17, 29.19, 27.71, 22.22. HRMS $(ES)^+$: m/z calculated for $[C_{24}H_{26}N_4NaO_2]^+$: 425.1948 $[M+Na]^+$; Found: 425.1949.

Compound 2

Compound 2 was synthesized according to the method reported in previous literature.[2] Briefly, a

solution of compound 1 (1 eq, 2.2 g, 5.47 mmol), nitromethane (20 eq, 5.87 mL, 109.3 mmol) and KOH (1.2 eq, 0.37 g, 6.54 mmol) in EtOH (10 mL) was heated at 60 ºC under reflux for 12 h. After cooling down to room temperature, the solvent was removed and residue obtained was acidified with 4 M HCl, and the crude product was extracted using EtOAc and washed with deionized water. The organic layers were collected and dried against Na_2SO_4. The crude product was purified by silica gel chromatography to obtain the final product (2.4 g, 94.5%). The obtained product (1 eq, 2.4 g, 5.18 mmol) and ammonium acetate (35 eq, 13.97 g, 181.2 mmol) were dissolved in butanol (50 mL). The mixture was heated at 130 ºC under reflux for 24 h. After cooling down, the residue was diluted with EtOAc followed by filtration. The crude product was extracted with EtOAc, and then washed with brine and ethanol. The organic layers were collected and dried against Na_2SO_4. The solvent was removed to give the product used for further synthesis without purification (0.92 g, 21.2%). The last product (1 eq, 0.32 g, 0.38 mmol) was dissolved in dry DCM (50 mL), treated with triethylamine (4 mL) and $BF_3 \cdot Et_2O$ (4 mL), and stirred under the nitrogen atmosphere for 48 h, followed by washing against water. The organic layers were collected and dried against Na_2SO_4. The crude product was purified by silica gel chromatography using DCM/hexane (v/v, 2:1) as eluent to give the final product as green metallic solid (0.30 g, 88.8%).^{1}H NMR (400 MHz, $CDCl_3$): δ 8.00 (d, J = 6.7 Hz, 4H), 7.60 (s, 4H), 6.96 (s, 4H), 6.72 (s, 2H), 4.10 (s, 4H), 3.53 (s, 4H), 3.27 (s, 8H), 2.77 (s, 8H), 1.99 (s, 12H). ^{13}C NMR (100 MHz, $CDCl_3$): δ 160.04, 155.93, 145.19, 143.92, 143.17, 131.15, 128.56, 125.57, 121.17, 120.78, 114.31, 64.53, 50.14, 48.26, 28.77, 28.04, 21.82. HRMS (APC)$^+$: m/z calculated for $[C_{50}H_{51}N_{11}O_2BF_2]^+$: 886.42828 $[M+H]^+$; Found: 886.42819.

BPY-Mal$_2$

Compound 2 (1 eq, 100 mg, 0.113 mmol) and DBCO-maleimide (2.4 eq, 115 mg, 0.27 mmol) were dissolved in dry DCM (30 mL), and the mixture was stirred at room temperature for 12 h. The solvent was removed, and the crude product was purified by silica gel chromatography using methanol/acetone/DCM (v/v/v, 1:6.5:50) as eluent to remove the impurity, and then using acetone/DCM (v/v, 1:2) as eluent to give the final product as dark blue metallic solid (0.138 g,

72.4%). ^{1}H NMR (400 MHz, $CDCl_3$): δ 8.00 (s, 2H), 7.89 (s, 2H), 7.60 (s, 6H), 7.43 (s, 7H), 7.16 (t, J = 9.7 Hz, 3H), 6.94 (s, 4H), 6.72 (s, 4H), 6.60 (s, 4H), 6.18 (s, 2H), 6.01 (s, 2H), 4.74 (s, 2H), 4.50 (s, 3H), 4.23 (s, 4H), 3.99 (s, 1H), 3.77 (s, 6H), 3.29 (s, 8H), 3.10 (s, 3H), 2.77 (s, 8H), 2.62 (s, 2H), 2.37 (s, 7H), 2.01 (s, 12H). ^{13}C NMR (150 MHz, $CDCl_3$): δ171.95, 170.53, 169.94, 159.98, 159.54, 156.05, 145.19, 143.99, 142.76, 139.90, 136.02, 134.92, 134.10, 131.79, 130.92, 129.84, 129.43, 128.59, 127.57, 127.14, 121.20, 114.87, 114.31, 114.13, 64.33, 54.51, 50.14, 45.17, 34.88, 31.35, 29.27, 28.04, 21.80. HRMS $(ES)^+$: m/z calculated for $[C_{100}H_{92}N_{17}O_{10}BF_2Na_2]^{2+}$: 892.8537$[M+2Na]^{2+}$; Found: 892.8551.

Section S2: Additional Supplementary Figures and Data

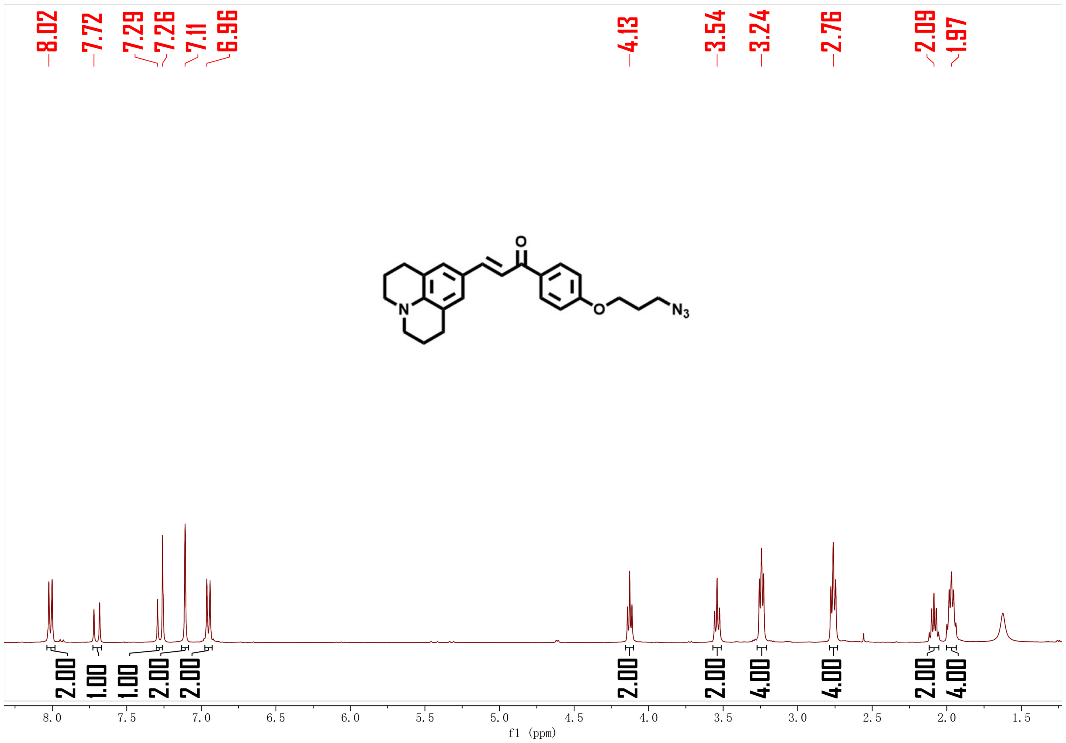

Supplementary Fig. 2. ^{1}H NMR spectrum of compound 1 in $CDCl_3$.

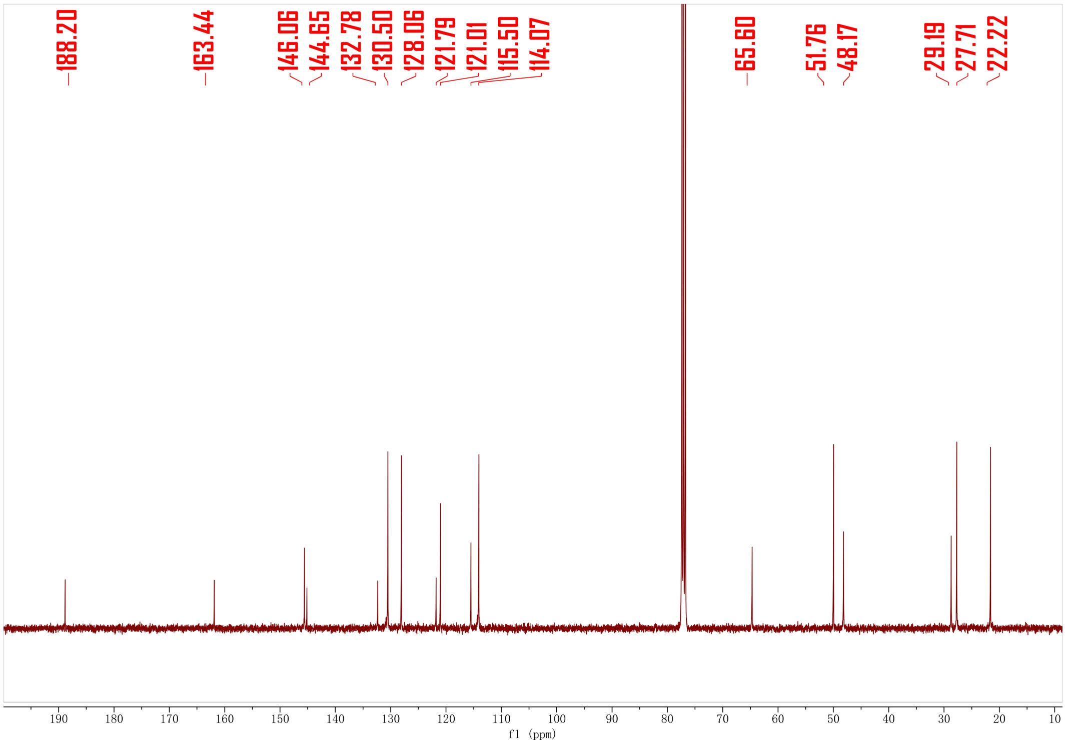

Supplementary Fig. 3. ^{13}C NMR spectrum of compound 1 in $CDCl_3$.

Elmt	Val.	Min	Max	Elmt	Val.	Min	Max	Elmt	Val.	Min	Max	Use Adduct
H	1	26	26	F	1	0	0	Br	1	0	0	H
C	4	24	24	P	3	0	0					Na
N	3	3	4	S	2	0	0					K
O	2	2	2	Cl	1	0	0					NH4

Error Margin (ppm):	5	DBE Range:	not fixed	Electron Ions:	both
HC Ratio:	0.0 - 100.0	Apply N Rule:	yes	Use MSn Info:	no
Max Isotopes:	all	Isotope RI (%):	1.00	Isotope Res:	10000
MSn Iso RI (%):	75.00	MSn Logic Mode:	AND	Max Results:	1000

Event#: 1 MS(E+) Ret. Time : 1.730 Scan# : 259

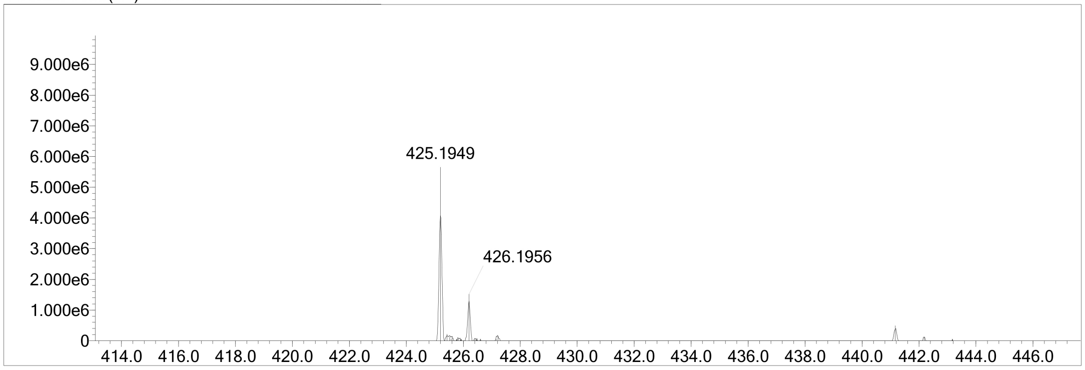

Supplementary Fig. 4. HRMS spectrum of compound 1.

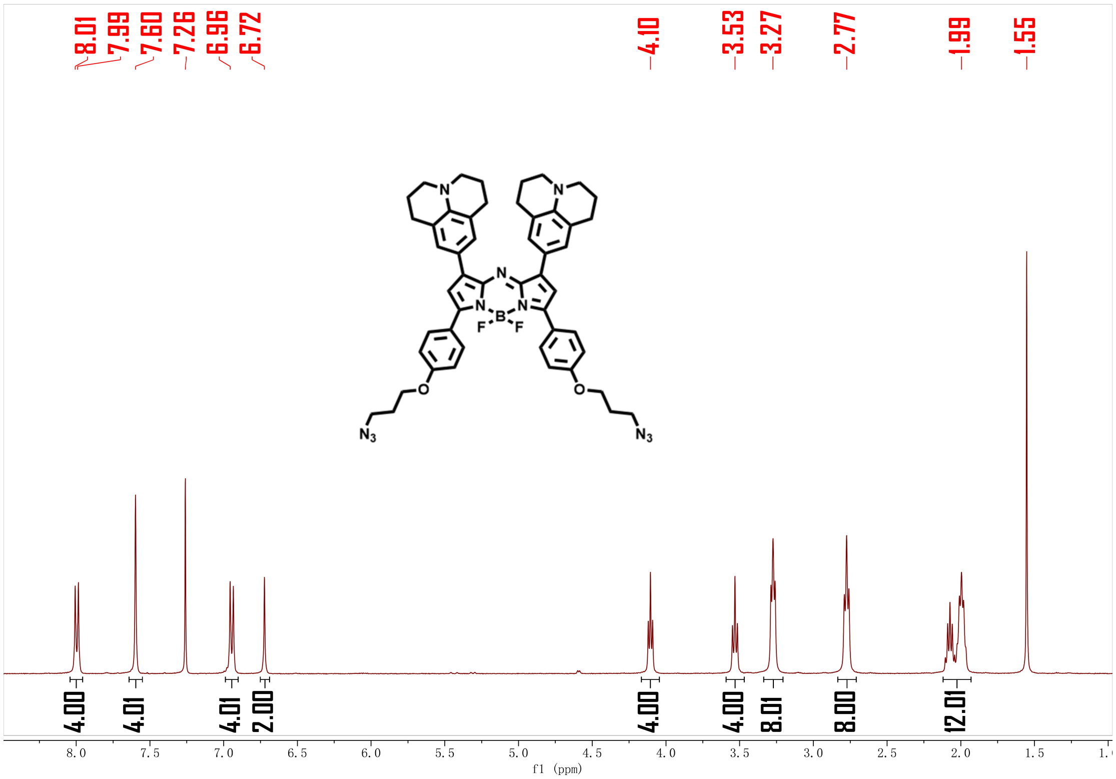

Supplementary Fig. 5. ¹H NMR spectrum of compound 2 in $CDCl_3$.

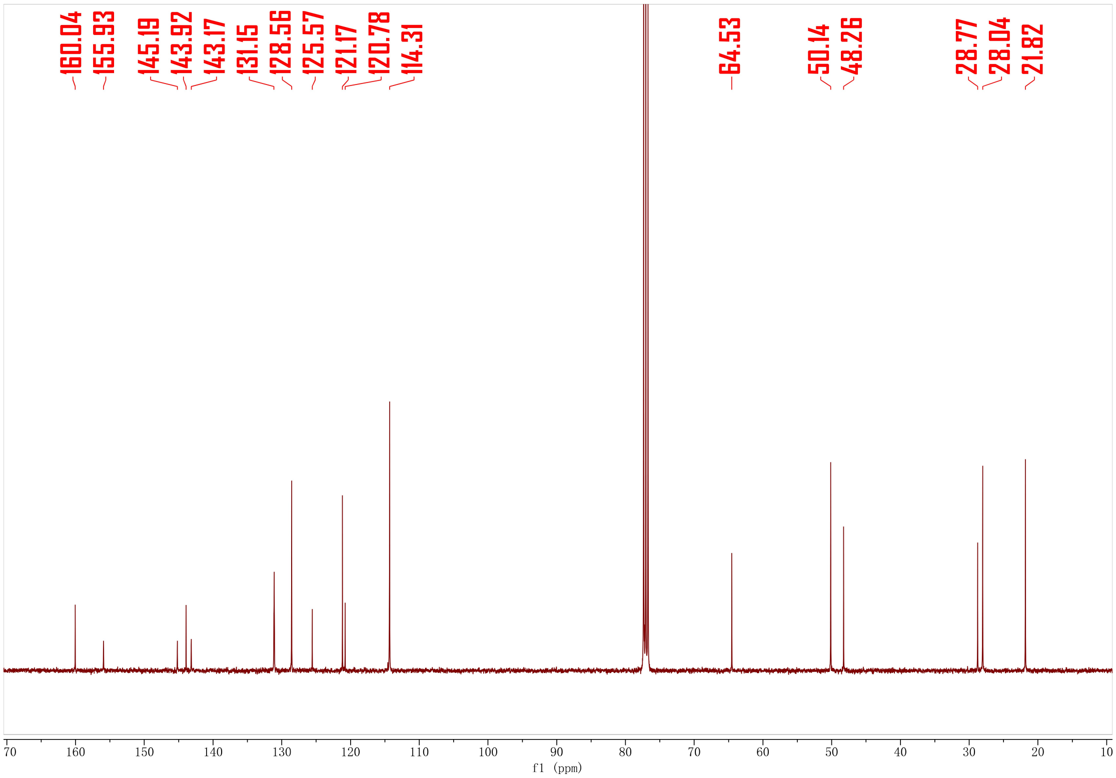

Supplementary Fig. 6. ^{13}C NMR spectrum of compound 2 in $CDCl_3$.

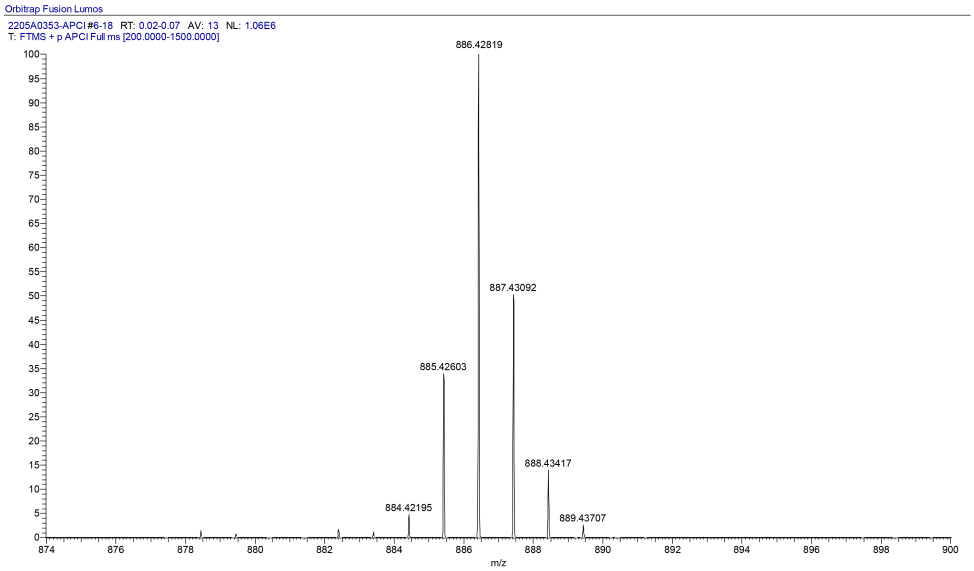

SPECTRUM - simulation :

m/z	Theo. Mass	Delta (ppm)	RDB equiv.	Composition
886.42819	886.42828	-0.11	30.5	C50 H51 O2 N11 B F2

Supplementary Fig. 7. HRMS spectrum of compound 2.

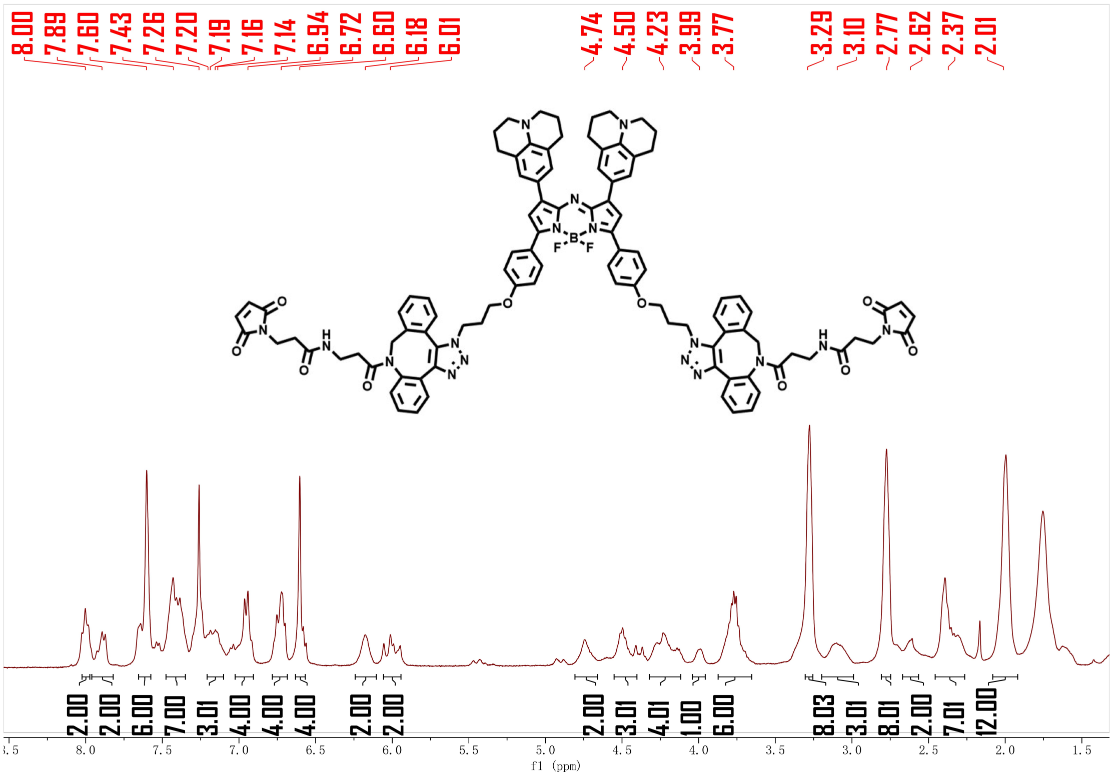

Supplementary Fig. 8. ^{1}H NMR spectrum of BPY-Mal_2 in $CDCl_3$.

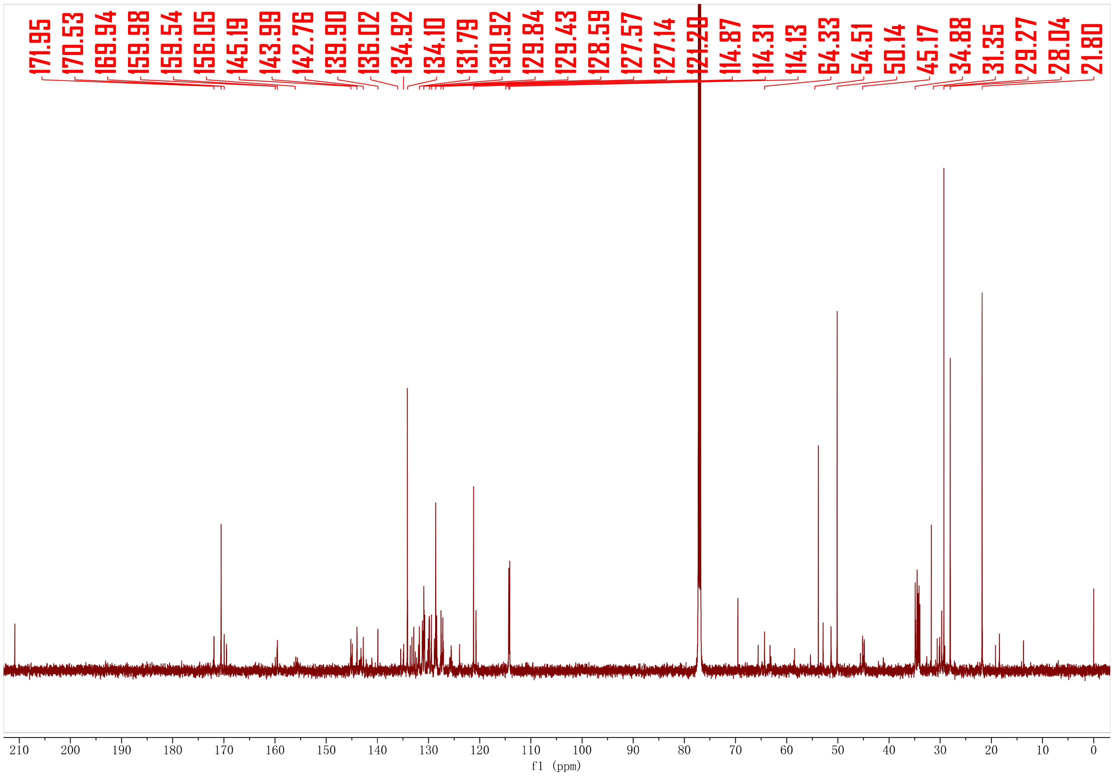

Supplementary Fig. 9. ^{13}C NMR spectrum of BPY-Mal_2 in $CDCl_3$.

Elmt	Val.	Min	Max	Elmt	Val.	Min	Max	Use Adduct
H	1	92	95	O	2	10	10	H
B	3	1	2	F	1	2	2	Na
C	4	100	100					K
N	3	17	17					NH4

Error Margin (ppm):	20	DBE Range:	not fixed	Electron Ions:	both
HC Ratio:	unlimited	Apply N Rule:	no	Use MSn Info:	no
Max Isotopes:	all	Isotope RI (%):	1.00	Isotope Res:	10000
MSn Iso RI (%):	75.00	MSn Logic Mode:	OR	Max Results:	100

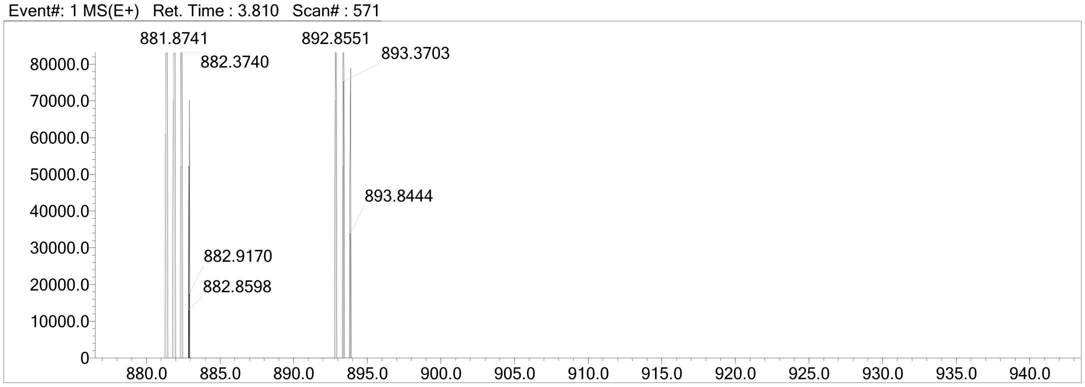

Supplementary Fig. 10. HRMS spectrum of BPY-Mal_2.

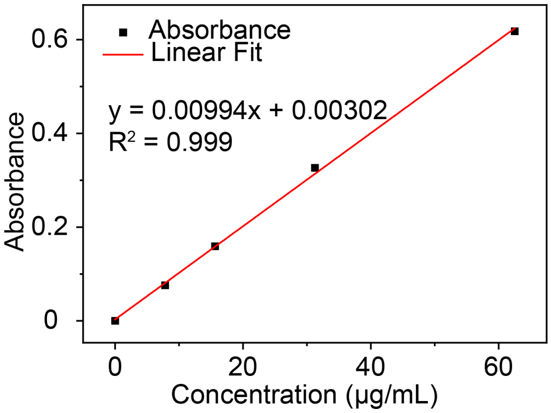

Supplementary Fig. 11. Linear relationship between absorbance at A_{808} and concentration of BPY-Mal_2 in water.

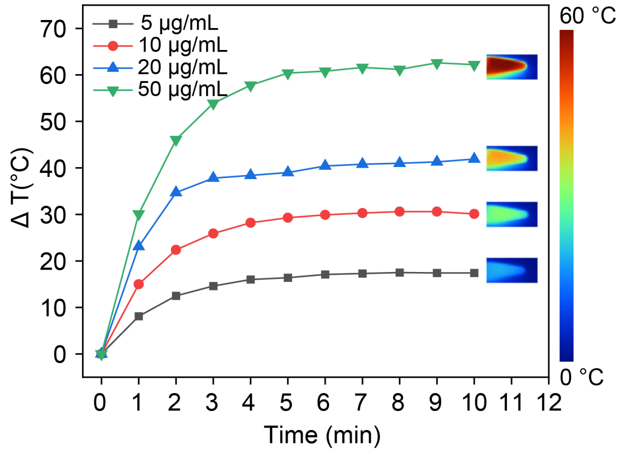

Supplementary Fig. 12. Photothermal properties of BPY with indicated concentrations (power density = 1 W/cm^2).

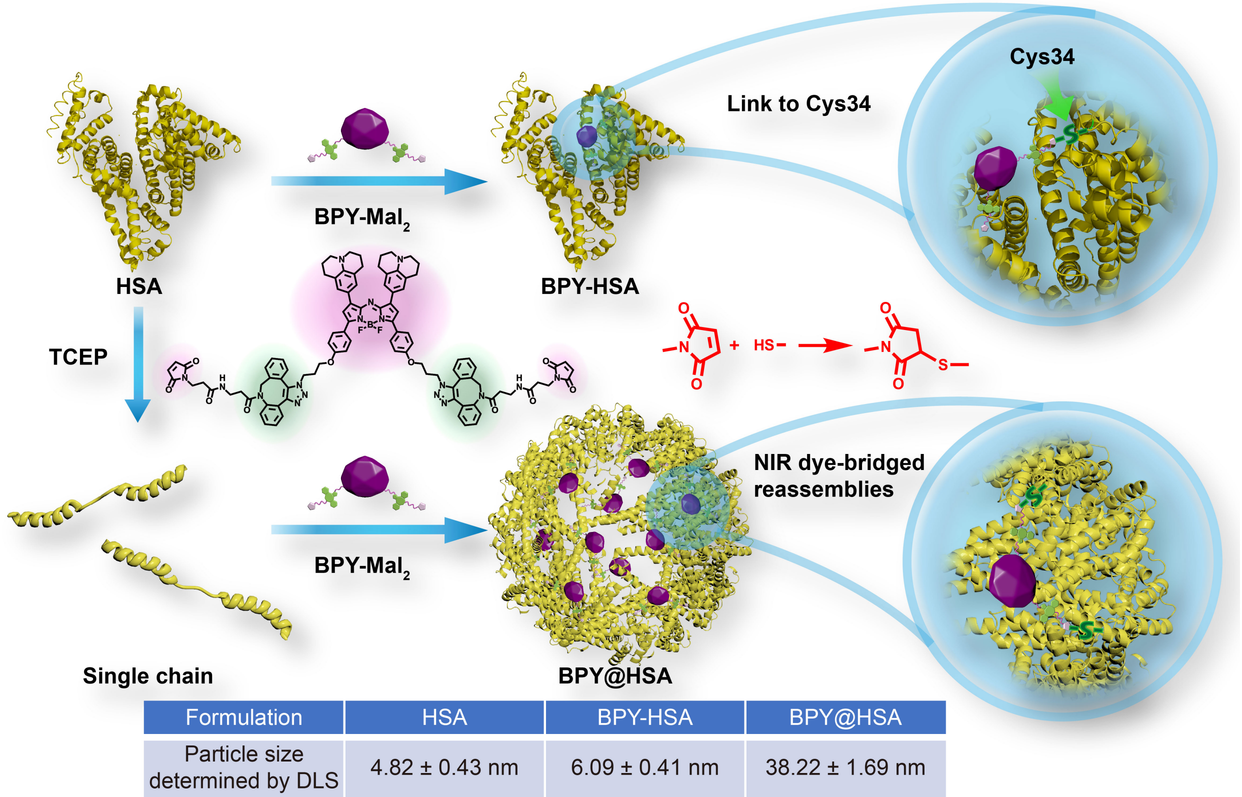

Formulation	HSA	BPY-HSA	BPY@HSA
Particle size determined by DLS	4.82 ± 0.43 nm	6.09 ± 0.41 nm	38.22 ± 1.69 nm

Supplementary Fig. 13. Comparation of BPY-HSA and BPY@HSA formulations. For BPY-HSA, each HSA protein owned only a free thiol group, and thus it could only afford one BPY-Mal_2 molecule. For BPY@HSA, all the disulfide bonds were deconstructed and then reconstructed by BPY-Mal_2 to form BPY-Mal_2-bridged nanoparticles with higher loading capacity.

Supplementary Note 1: Discussion for Supplementary Fig. 13

The assembly of multiple polypeptide chains is driven by the Michael reaction between the free thiols on polypeptide chains and maleimide groups on the BPY-Mal_2 (the red equation in Supplementary Fig. 13), which spontaneously forms covalent bonds between thiols and BPY-Mal_2. In detail, the BPY-Mal_2 was directly loaded to BPY-HSA formulation by the spontaneous reaction between thiols (on the Cys34 of the HSA) and maleimide groups (on the BPY-Mal_2 molecules), which preserved the natural structure of the HSA protein. For BPY@HSA formulations, the disulfide bonds linking the two polypeptide chains were cleaved to thiols by TCEP, and the thiols were further reacted with the maleimide groups of bi-maleimide functionalized BPY-Mal_2 to bridge the polypeptide chains to form reassemblies. During the bridging process, the hydrophilic polypeptide chains were linked by hydrophobic BPY-Mal_2 molecules to reassemble BPY@HSA nanoparticles with the structure of hydrophilic polypeptide shell and hydrophobic drug loaded core.

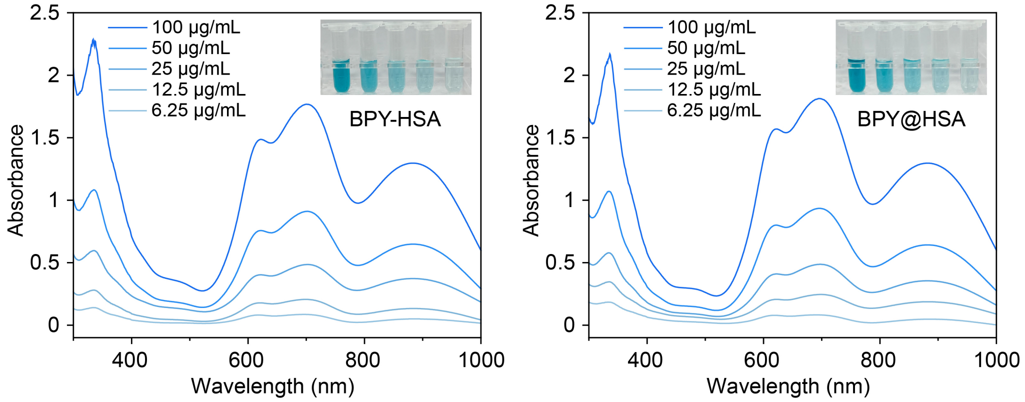

Supplementary Fig. 14. Digital photos and absorbance of BPY-HSA and BPY@HSA with indicated concentrations.

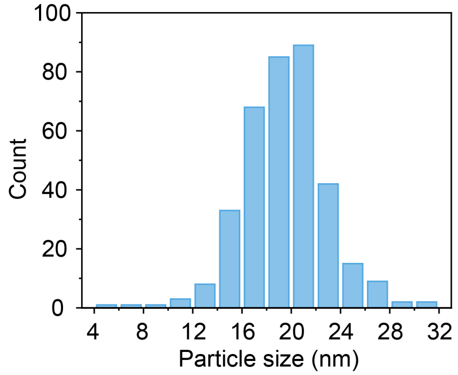

Supplementary Fig. 15. Size distribution histogram counted from TEM image in Fig. 2a.

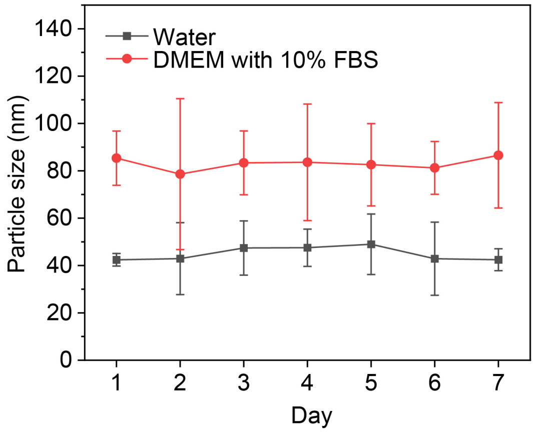

Supplementary Fig. 16. Size stability of BPY@HSA in water and DMEM complete medium containing 10% FBS. The particle size increased in DMEM with 10% FBS, indicating that the protein corona may be formed on the surface of BPY@HSA by adsorbing proteins like immunoglobulin, apolipoprotein, and albumin. The size presented negligible changes in 7 days, indicating that such interaction between proteins and nanoparticles was stable.

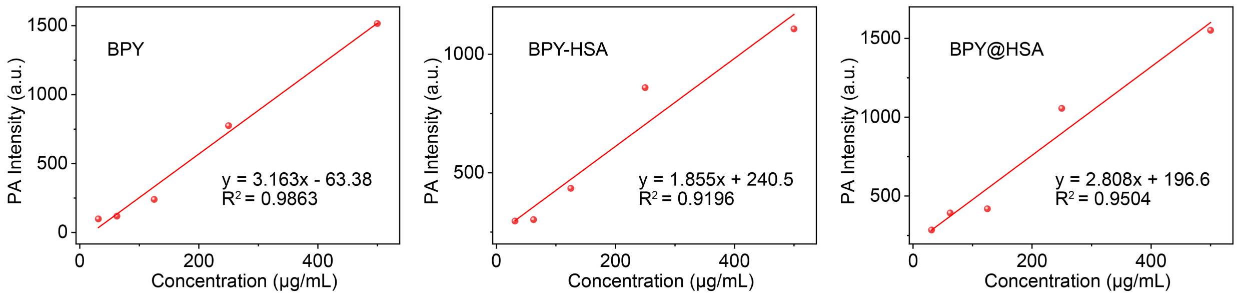

Supplementary Fig. 17. Linear relationships between PA intensity and concentrations of BPY, BPY-HSA and BPY@HSA.

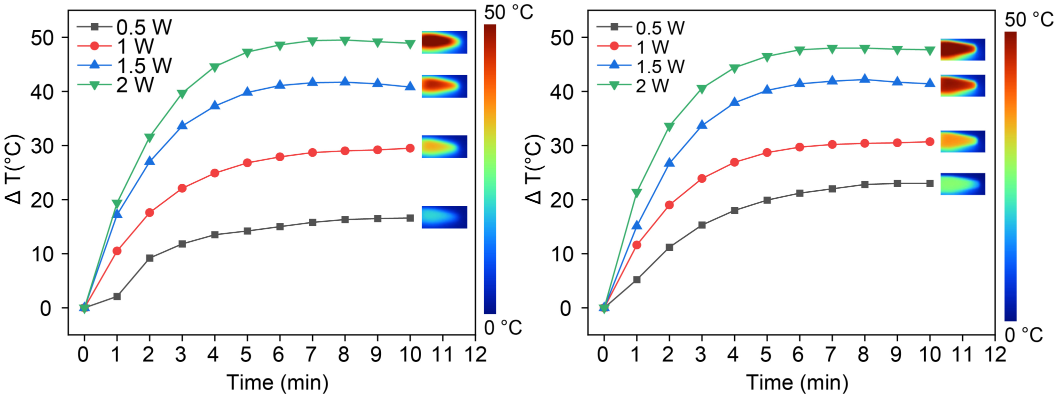

Supplementary Fig. 18. Photothermal temperature elevation curves of (left) BPY-HSA and (right) BPY@HSA under 808 nm laser irradiation at indicated power (concentration = 20 μg/mL).

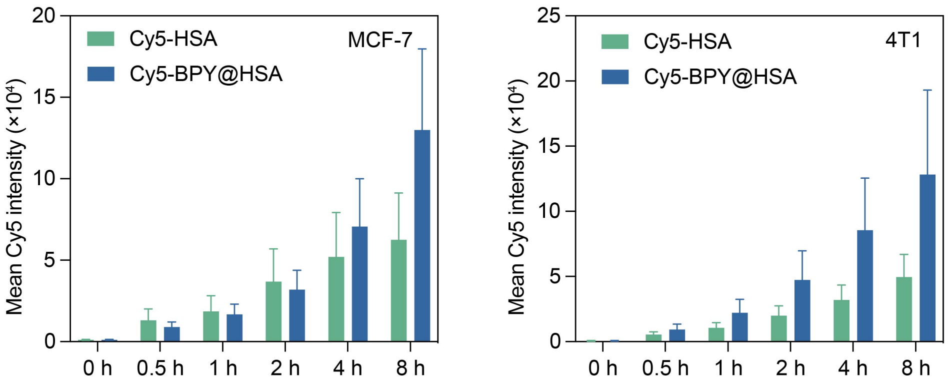

Supplementary Fig. 19. Mean Cy5 intensity of cellular uptake studies for Cy5-HSA and Cy5-BPY@HSA formulations on MCF-7 and 4T1 cell lines.

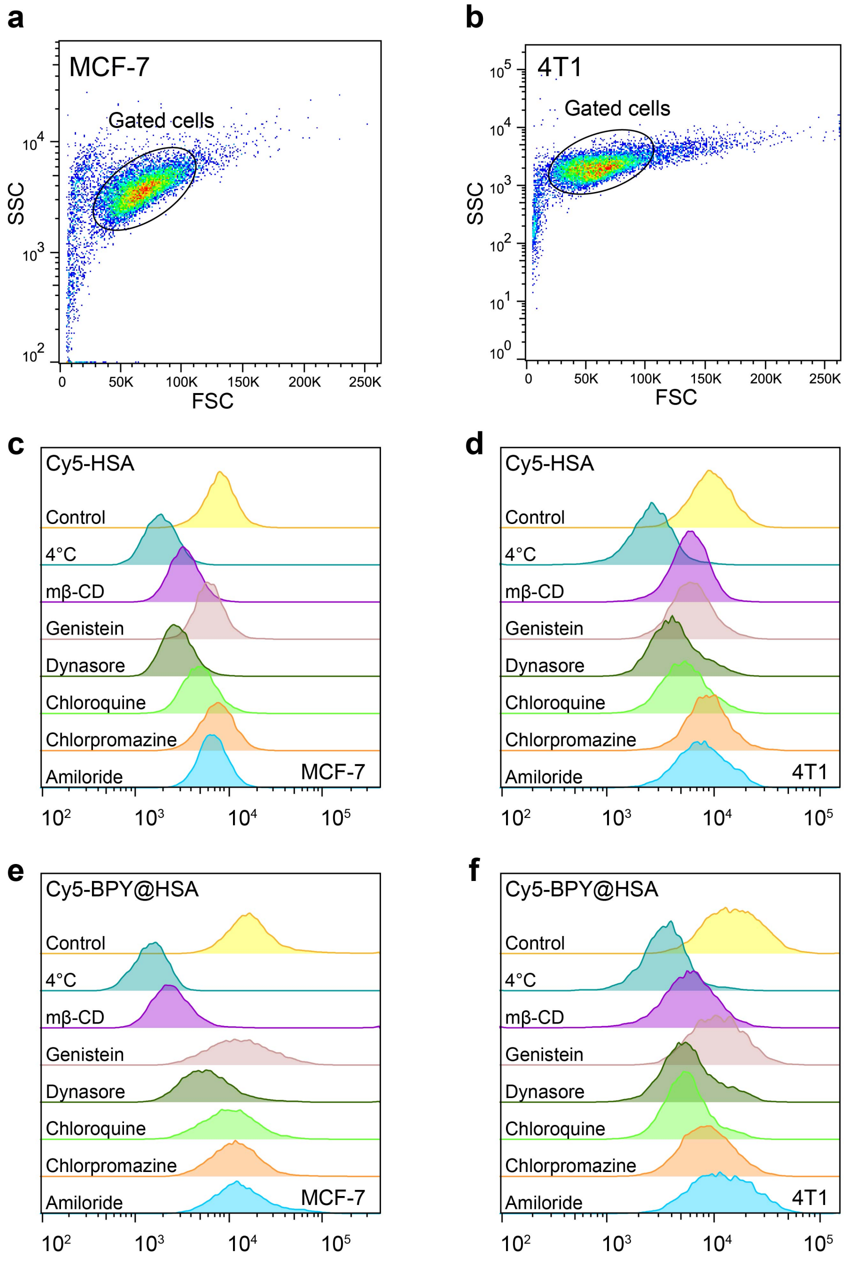

Supplementary Fig. 20. Gating strategies and representative fluorescence histograms. Gating strategies for cellular uptake study on **a** MCF-7 and **b** 4T1 cell lines. Representative fluorescence histograms of **c, e** MCF-7 and **d, f** 4T1 cells administrated with 5 μg/mL Cy5-HSA and Cy5-BPY@HSA at different conditions, respectively.

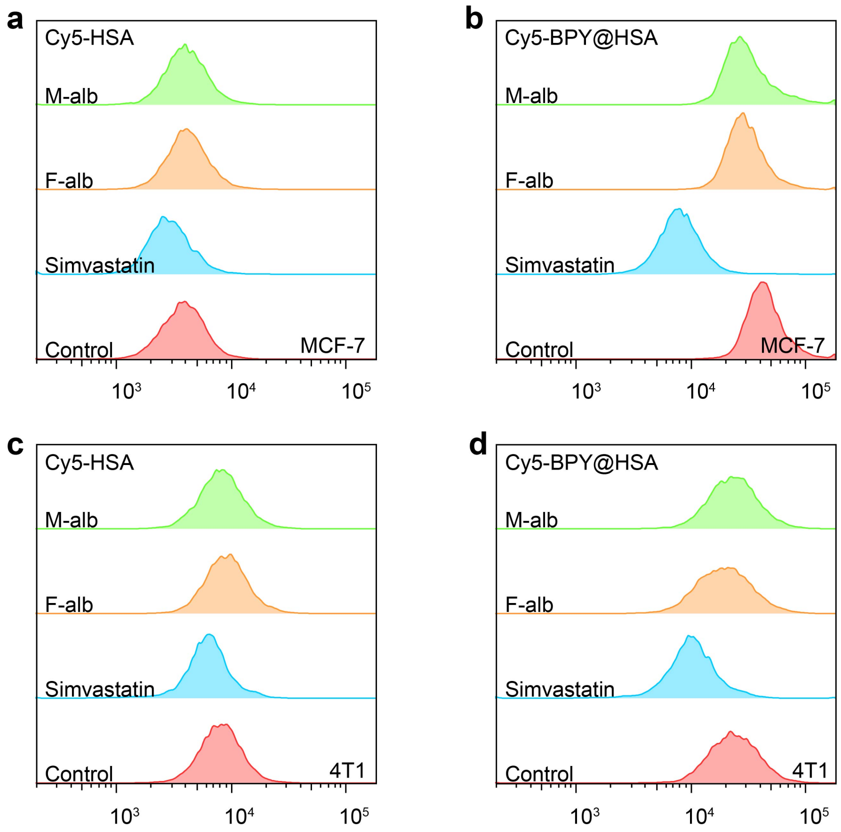

Supplementary Fig. 21. Representative fluorescence histograms. Representative fluorescence histograms of the investigation to receptor-mediated endocytosis on **a**, **b** MCF-7 and **c**, **d** 4T1 cells administrated with 5 μg/mL Cy5-HSA and Cy5-BPY@HSA at different conditions, respectively.

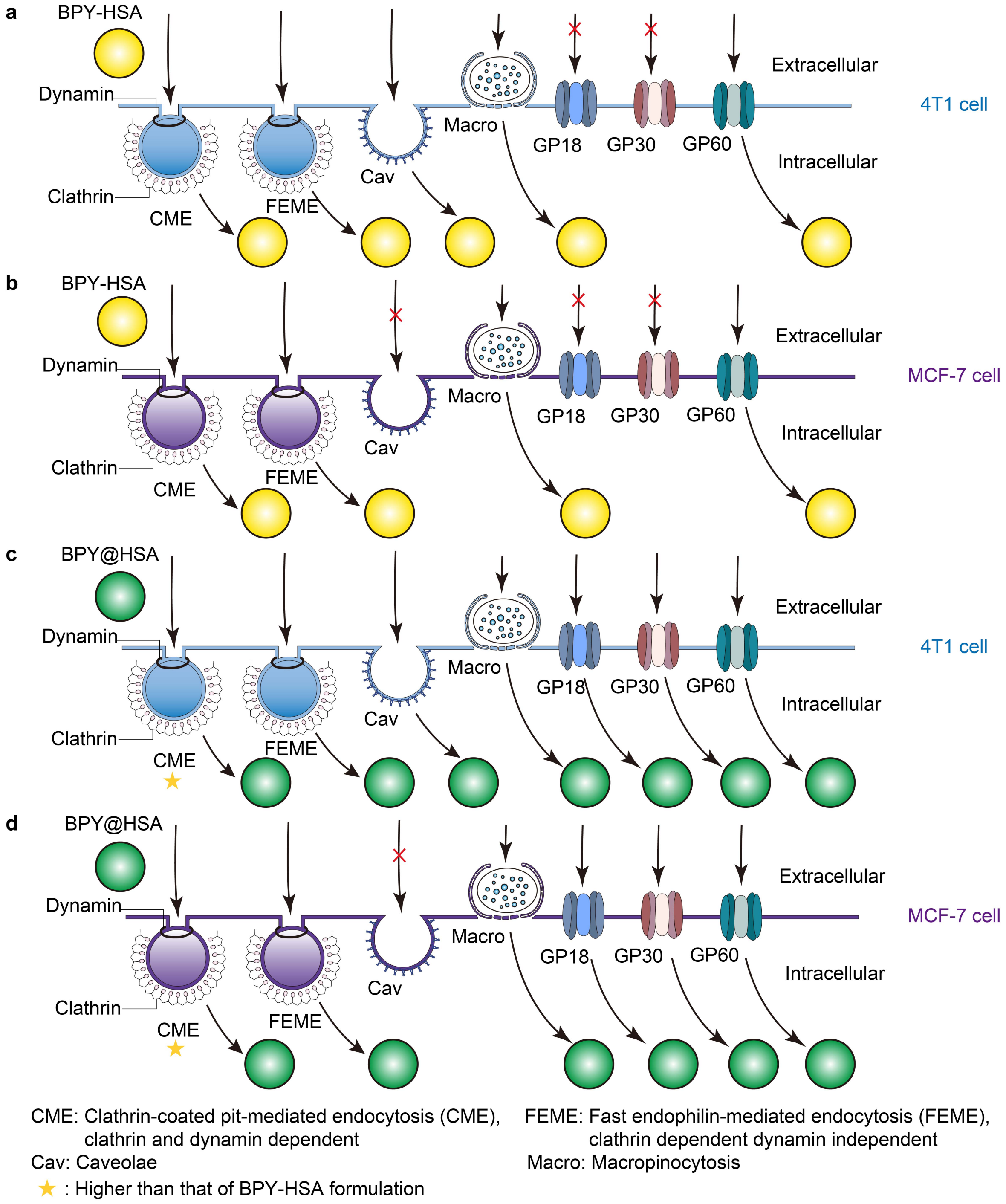

Supplementary Fig. 22. Endocytosis mechanisms. Schematic illustration for the endocytosis mechanisms of BPY-HSA in **a** 4T1 and **b** MCF-7 cells, and BPY@HSA in **c** 4T1 and **d** MCF-7 cells. BPY@HSA presented superior endocytosis performance than BPY-HSA since it involves more uptake pathways and receptor interactions.

Supplementary Note 2: Discussion for cellular uptake mechanisms and receptor interactions

Cy5-HSA presented different uptake behavior on MCF-7 and 4T1 cell lines. As there is no molecular machinery of caveolae in MCF-7 cell line[3,4], the uptake efficacy decreasing in Simvastatin revealed that the MCF-7 cell line depended on clathrin-independent carriers (CLIC) rather than caveolin, while the 4T1 cell line depended on both CLIC and caveolin. Therefore, the cellular uptake of MCF-7 depended on clathrin-mediated endocytosis (CME), clathrin-independent/dynamin-dependent endocytosis (FEME), CLIC, and macropinocytosis, and that of 4T1 depended on CME, FEME, caveolin, and CLIC. This might be the reason why Cy5-HSA presented higher uptake level in 4T1 cell line.

Cy5-BPY@HSA interacted with different receptors on MCF-7 and 4T1 cell lines. The major receptors mediating cellular uptake of the HSA and HSA-based materials are gp60, gp18, and gp30. As HSA is known for gp60 receptor-mediated endocytosis, level of gp60 receptor-mediated endocytosis to Cy5-BPY@HSA formulation could be estimated by the difference in the decline rates of simvastatin and mβ-CD between the two cell lines. Compared with Cy5-HSA formulation, Cy5-BPY@HSA formulation presented a significant inhibition on simvastatin (79.6% versus 23.3%) but no crucial inhibition on mβ-CD (40.5% versus 31.8%) in MCF-7 cell line, demonstrating that the uptake of Cy5-HSA formulation in MCF-7 cells depended more on gp60 whereas Cy5-BPY@HSA formulation not. As a contrast, Cy5-BPY@HSA formulation presented moderate inhibition on simvastatin (77.3% versus 42.1%) and mβ-CD (59.9% versus 40.3%), implying that both Cy5-HSA and Cy5-BPY@HSA formulations depended on gp60 in 4T1 cells. Therefore, Cy5-BPY@HSA formulation depended on gp18 and gp30 receptor-mediated endocytosis in MCF-7 cell line, and it depended more on gp18 and gp60 receptor-mediated endocytosis in 4T1 cell line.

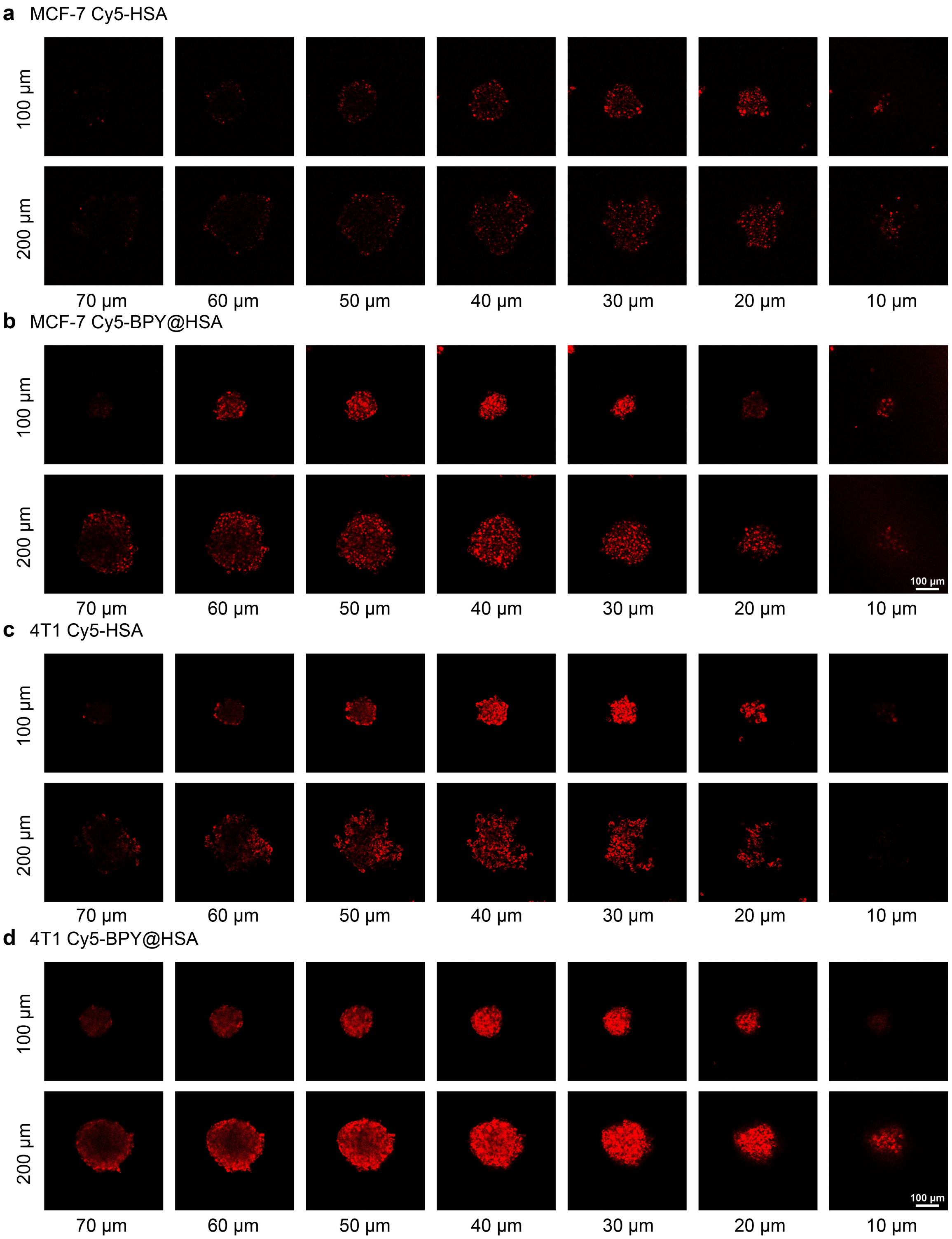

Supplementary Fig. 23. Z-stack CLSM images. Z-stack CLSM images of **a,b** MCF-7 and **c,d** 4T1 3D tumor spheroids with different treatments indicated.

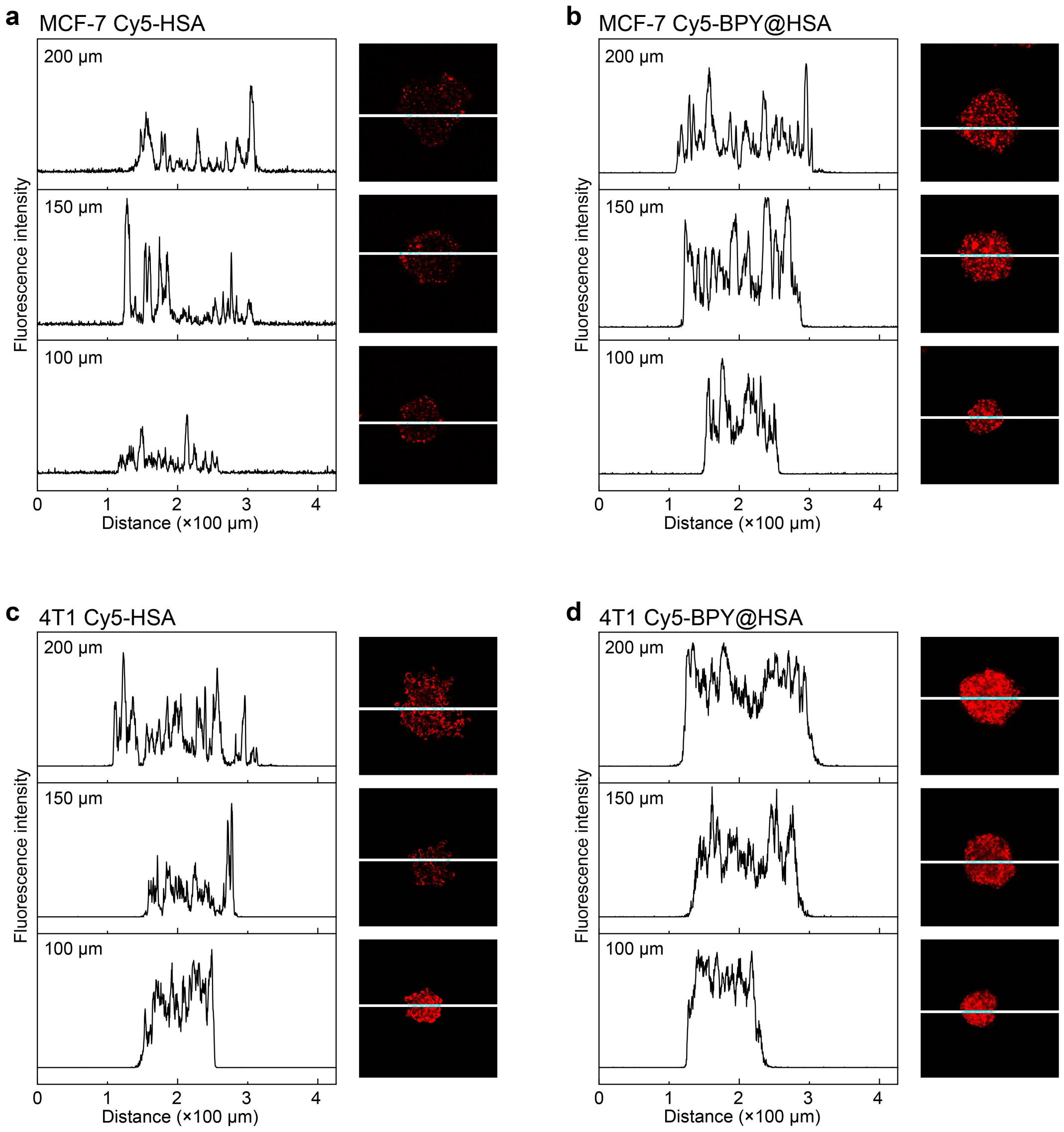

Supplementary Fig. 24. Fluorescence intensities. a-d Fluorescence intensities of lined region on Z-stack CLSM images for infiltration analysis in tumor spheroids with different treatments indicated.

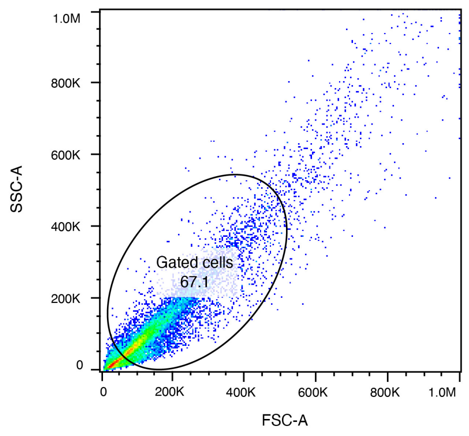

Supplementary Fig. 25. Gating strategies for apoptosis analysis of MCF-7 cells.

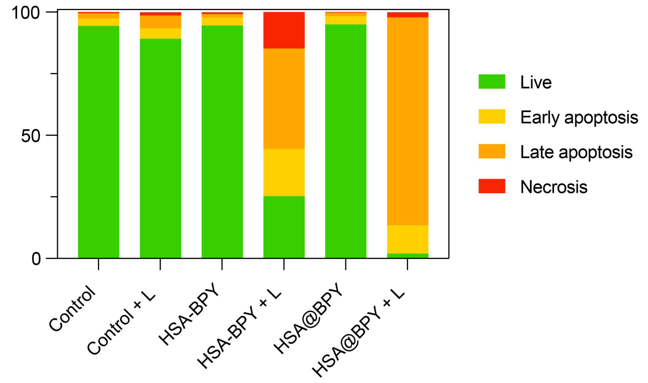

	Control	Control + L	BPY -HSA	BPY -HSA + L	BPY@HSA	BPY@HSA + L
Live (%)	94.4	89.2	94.6	25.3	95	2.01
Early apoptosis (%)	3.03	4.18	3.13	19.1	3.24	11.4
Late apoptosis (%)	2.09	5.24	1.49	40.8	1.53	84.4
Necrosis (%)	0.51	1.34	0.74	14.8	0.23	2.13

Supplementary Fig. 26. Percentage analysis of MCF-7 apoptosis with different treatments indicated.

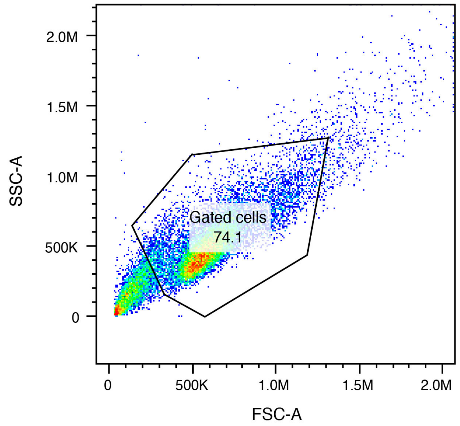

Supplementary Fig. 27. Gating strategies for apoptosis analysis of 4T1 cells.

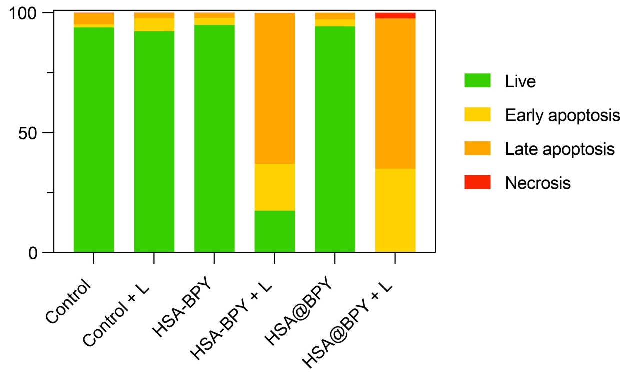

	Control	Control + L	BPY -HSA	BPY -HSA + L	BPY@HSA	BPY @HSA + L
Live (%)	93.9	92.3	94.9	17.5	94.3	0.26
Early apoptosis (%)	1.28	5.46	2.92	19.4	2.92	34.7
Late apoptosis (%)	4.56	2.2	2.03	62.9	2.77	62.6
Necrosis (%)	0.31	0.075	0.15	0.18	0.039	2.43

Supplementary Fig. 28. Percentage analysis of 4T1 apoptosis with different treatments indicated.

Supplementary Note 3: Discussion for Supplementary Fig. 26 and 28

The apoptosis rates of each treatment were given in Supplementary Fig. 26 and 28, and the BPY@HSA+L groups (95.8% for MCF-7 and 97.3% for 4T1) exhibited higher apoptosis rates than that of BPY-HSA+L groups (59.9% for MCF-7 and 82.3% for 4T1). The cellular uptake results (Fig. 3a-d) revealed that both cell lines presented enhanced uptake to BPY@HSA, and the 4T1 also showed preferred uptake to BPY-HSA than MCF-7. Meanwhile, the order of uptake efficacies of these groups (Cy5-BPY@HSA on 4T1 > Cy5-BPY@HSA on MCF-7 > Cy5-HSA on 4T1 > Cy5-HSA on MCF-7) were corresponded with the order of apoptosis rates. Therefore, we can conclude that the intracellular irradiated PTAs could generate higher cytotoxicity than that of extracellular PTAs.

Supplementary Note 4: Discussion for Supplementary Fig. 29

For the MCF-7 xenografted tumor model, the mice were positioned on their side under anesthesia to ensure clear observation of the tumor. As shown in Supplementary Fig. 29, the Cy5-HSA formulation exhibited systemic distribution in regions with high blood flow, which was probably due to that albumin is the major component of the blood. On the other hand, Cy5-BPY@HSA accumulated preferentially in the tumor, liver, and kidney. In particular, the Cy5-BPY@HSA initially entered the liver and reached their maximum concentration at 6 h before gradually being excreted through the kidneys and gastrointestinal (GI) tract, which suggested that the liver and kidneys were the primary organs responsible for the metabolism and excretion of the Cy5-BPY@HSA. In the MCF-7 xenograft tumor model, the Cy5-BPY@HSA showed greater accumulation in the tumor with an enhanced trend over a 24-hour period, which was related to the particle size differences (Supplementary Fig. 13) and tumor uptake differences (Supplementary Fig. 22) between the two formulations. However, Cy5-HSA demonstrated minimal accumulation in the tumor as the fluorescence signal was indistinguishable from the systemic background. Therefore, we analyzed the extent of accumulation through changes over time and *ex vivo* imaging.

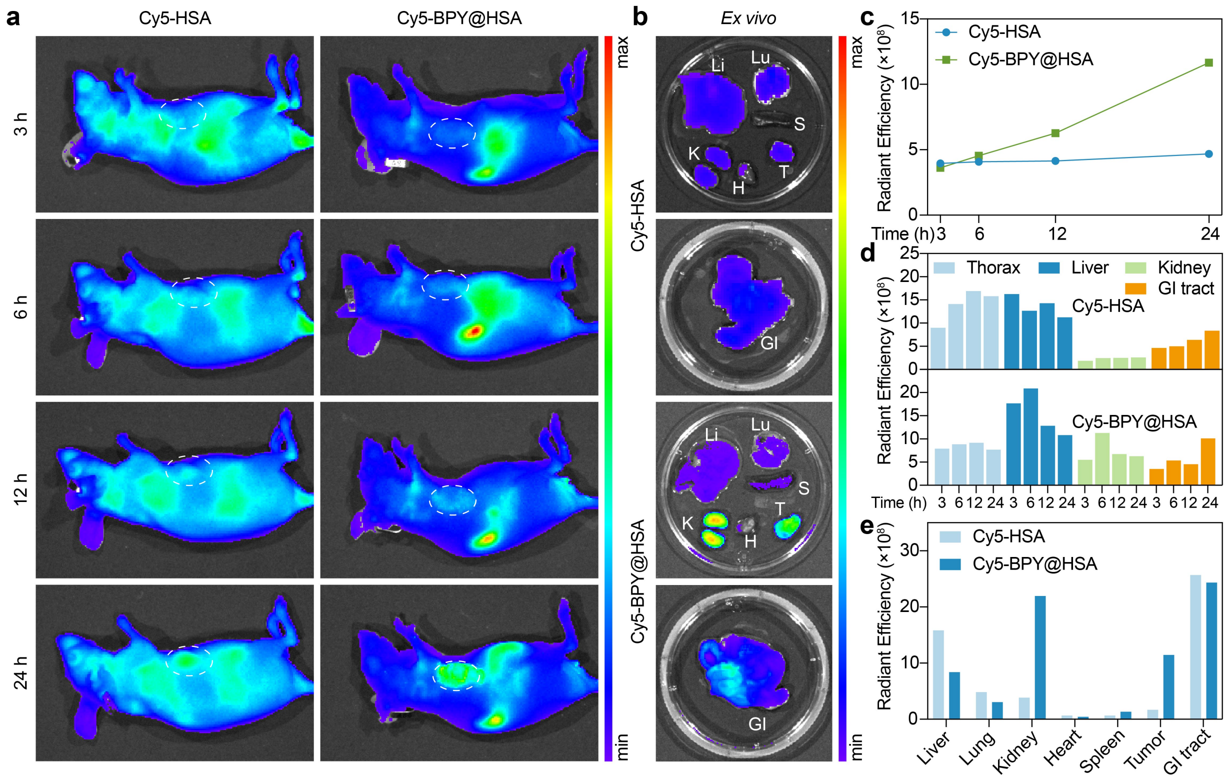

Supplementary Fig. 29. Biodistribution investigation of the Cy5-HSA and Cy5-BPY@HSA formulations on MCF-7 xenografted tumor models. a *In vivo* imaging at indicated time points post intravenous injection of Cy5-HSA and Cy5-BPY@HSA (white cycles indicate tumor areas). **b** *ex vivo* imaging of harvested major organs and tumors at 24 h post intravenous injection of Cy5-HSA and Cy5-BPY@HSA. Radiant efficacy of **c** tumor area, **d** selected areas against time, and **e** *ex vivo* organs and tissues (n = 1).

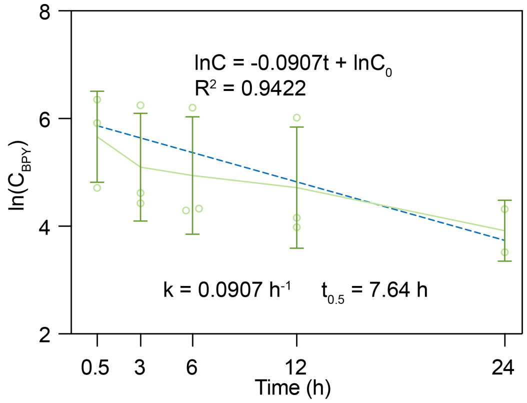

Supplementary Fig. 30. Blood concentration of BPY@HSA changes against time (n = 3).

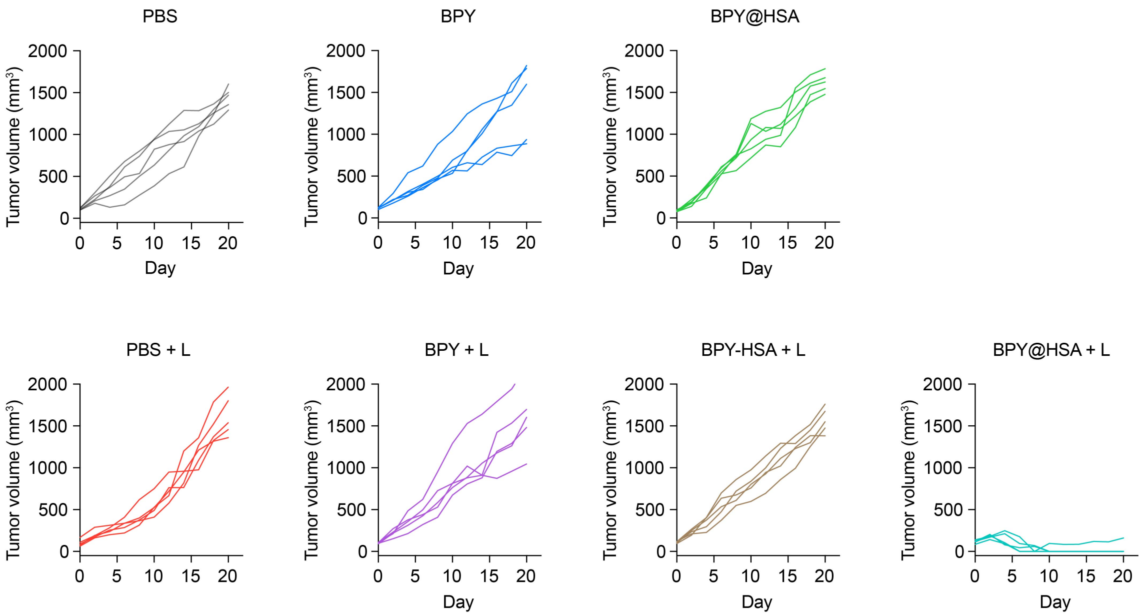

Supplementary Fig. 31. Tumor growth curves of MCF-7 tumor models (n = 5) with different treatments indicated.

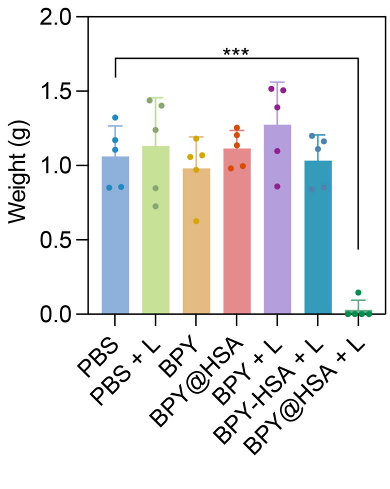

Supplementary Fig. 32. Harvested tumor weights of MCF-7 tumor models (n = 5) with different treatments indicated.

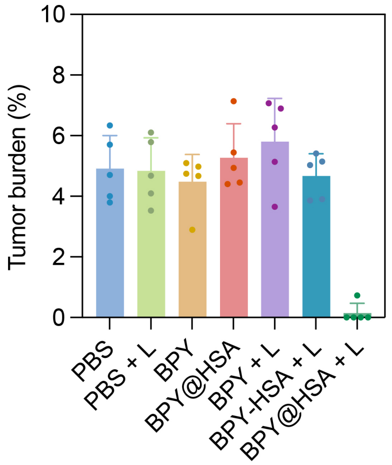

Supplementary Fig. 33. Tumor burden at the end of treatment on MCF-7 tumor models (n = 5) with different treatments indicated.

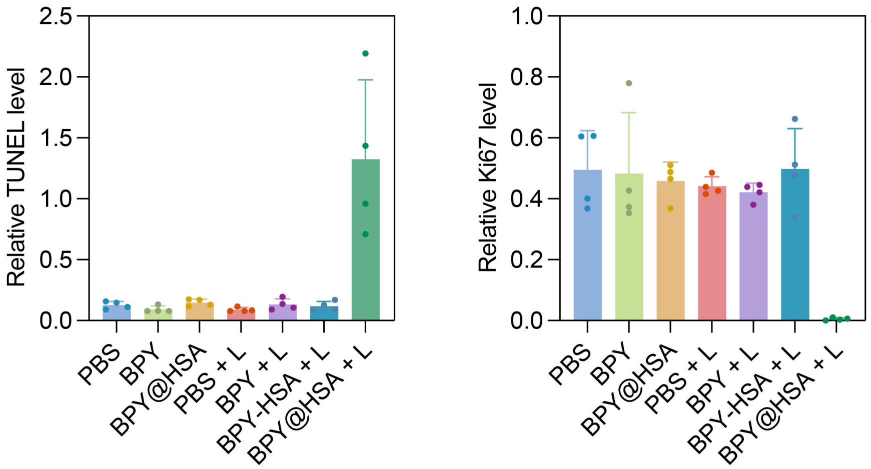

Supplementary Fig. 34. MFI analysis to TUNEL and Ki67 tumor slides of MCF-7 models with different treatments indicated (n = 4).

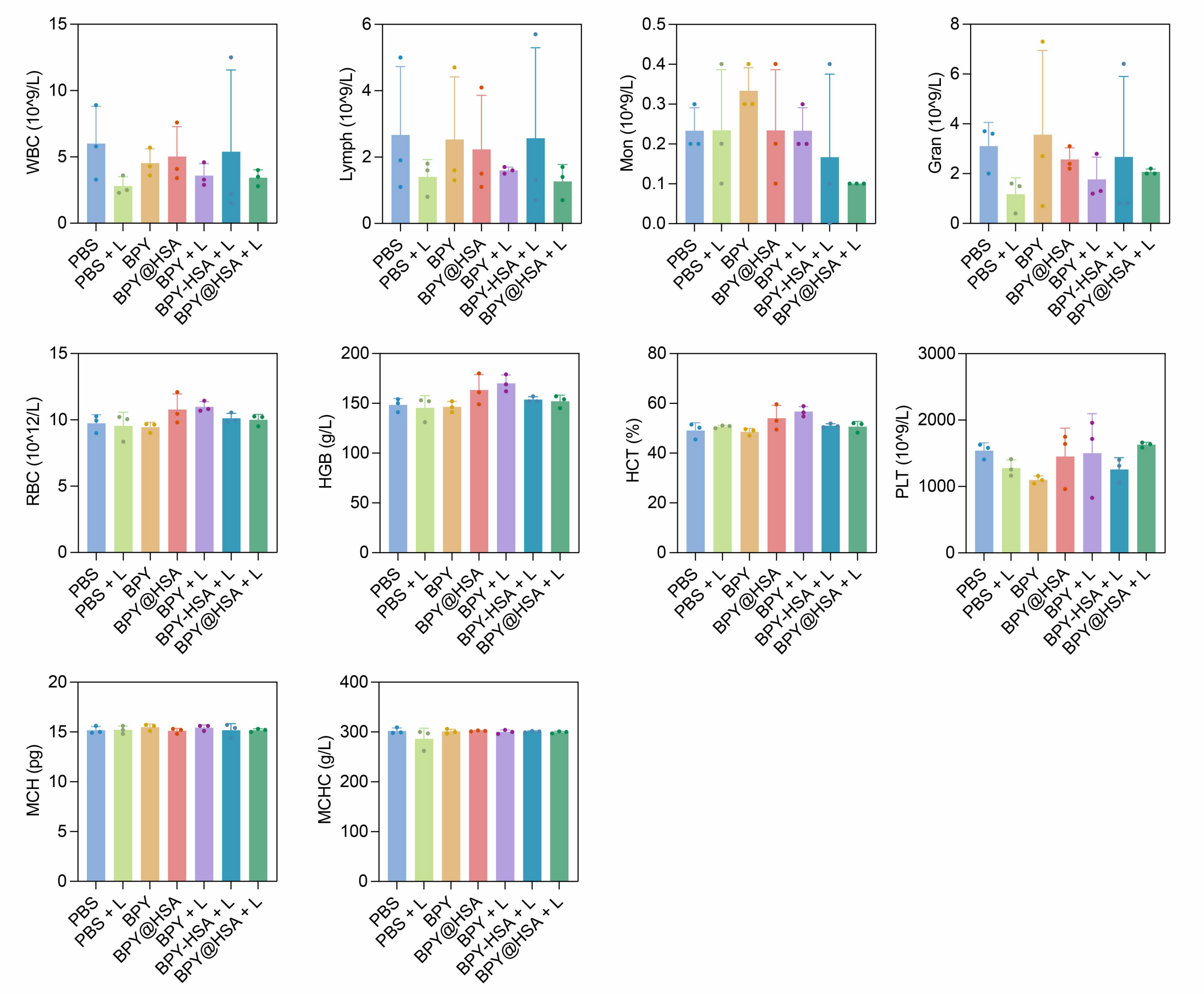

Supplementary Fig. 35. Routine blood analysis of different treatment groups (n = 3).

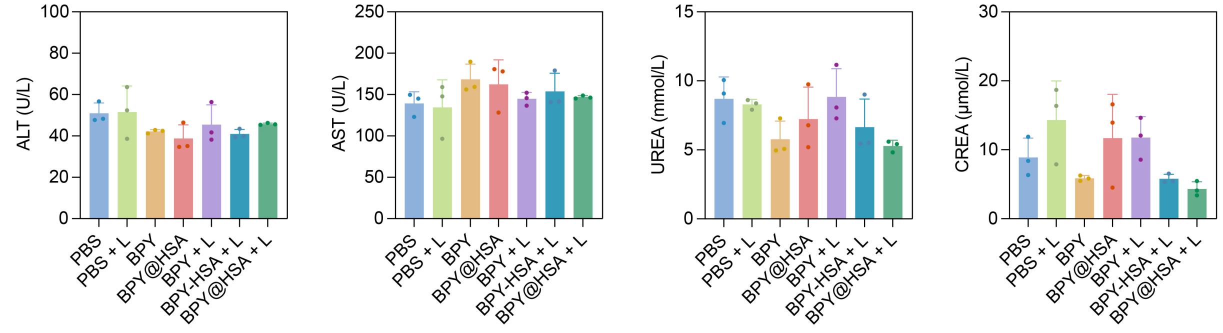

Supplementary Fig. 36. Blood biochemistry assays of different treatment groups (n = 3).

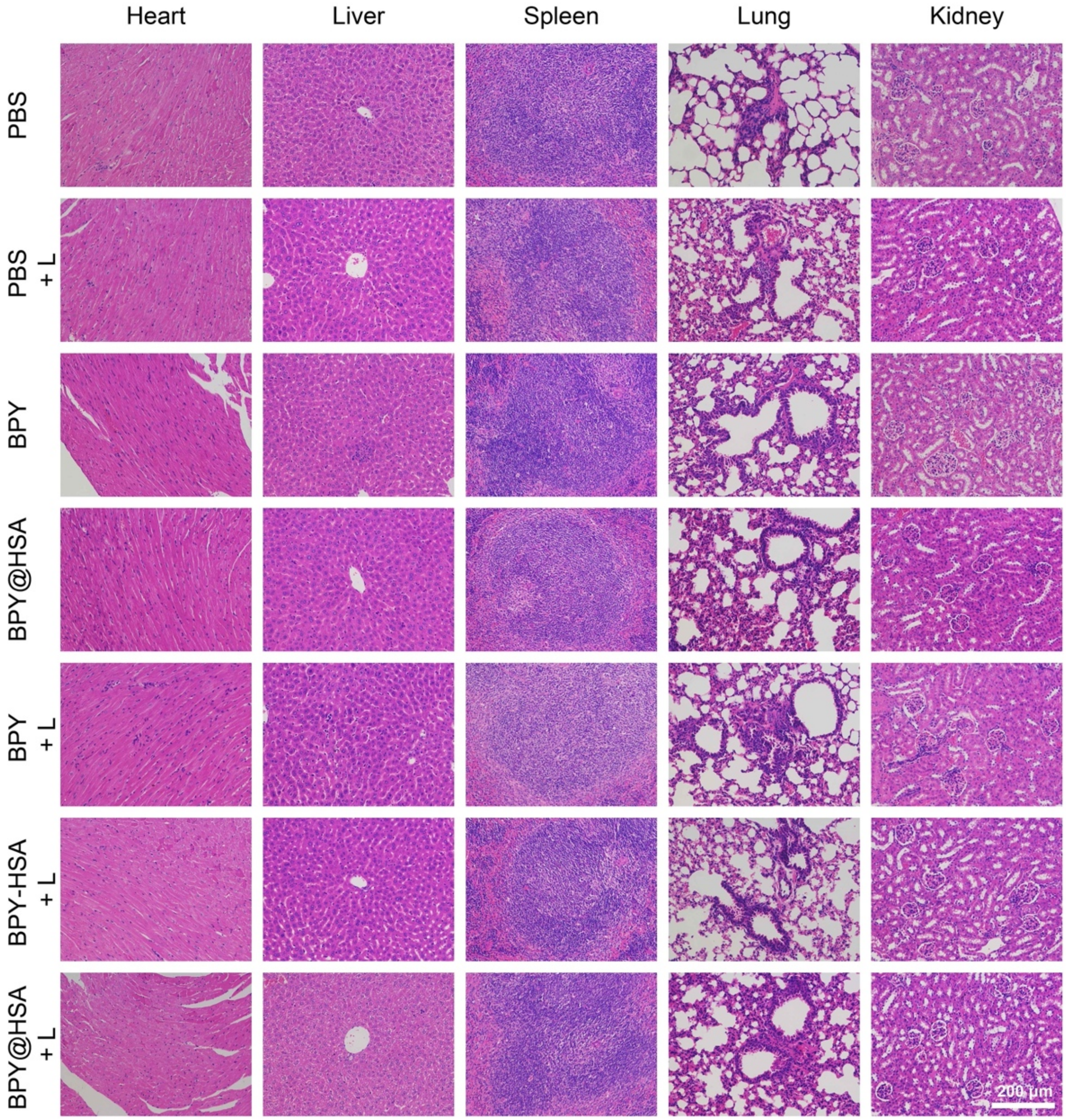

Supplementary Fig. 37. Representative H&E staining slides of major organs harvested from MCF-7 model with different treatments indicated. No obvious histological damages were founded in each treatment group.

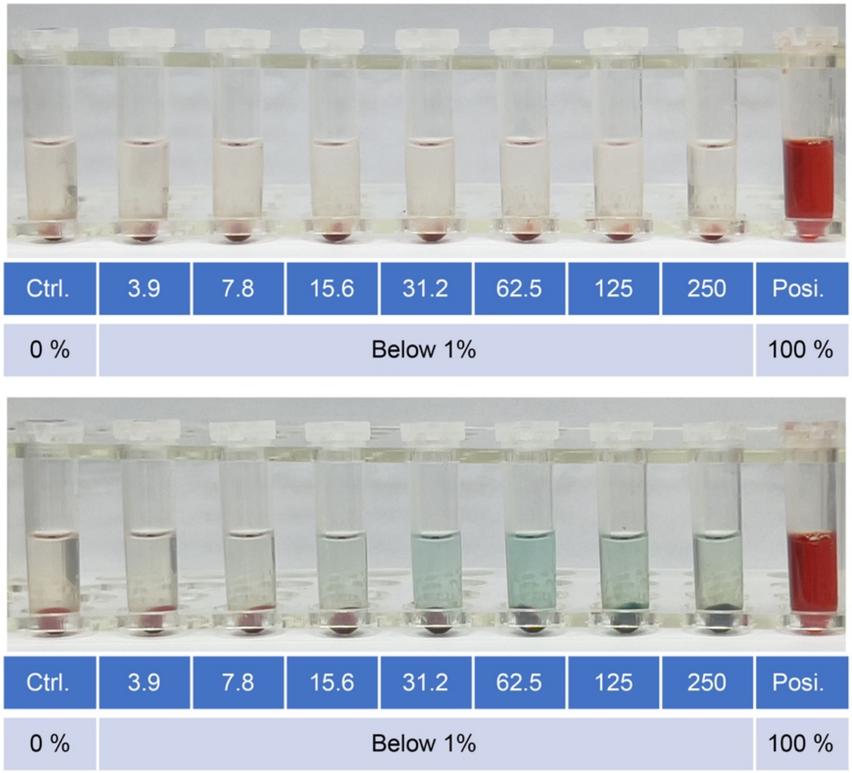

Supplementary Fig. 38. Hemolysis tests for HSA and BPY@HSA. Ctrl. represents negative control group, Posi. represents positive control group, and the concentration of HSA or BPY@HSA is ranged from 3.9 to 250 μg/mL. The hemolysis rates of all tested groups were below 1%, indicating the good biosafety of the BPY@HSA nanoplatform.

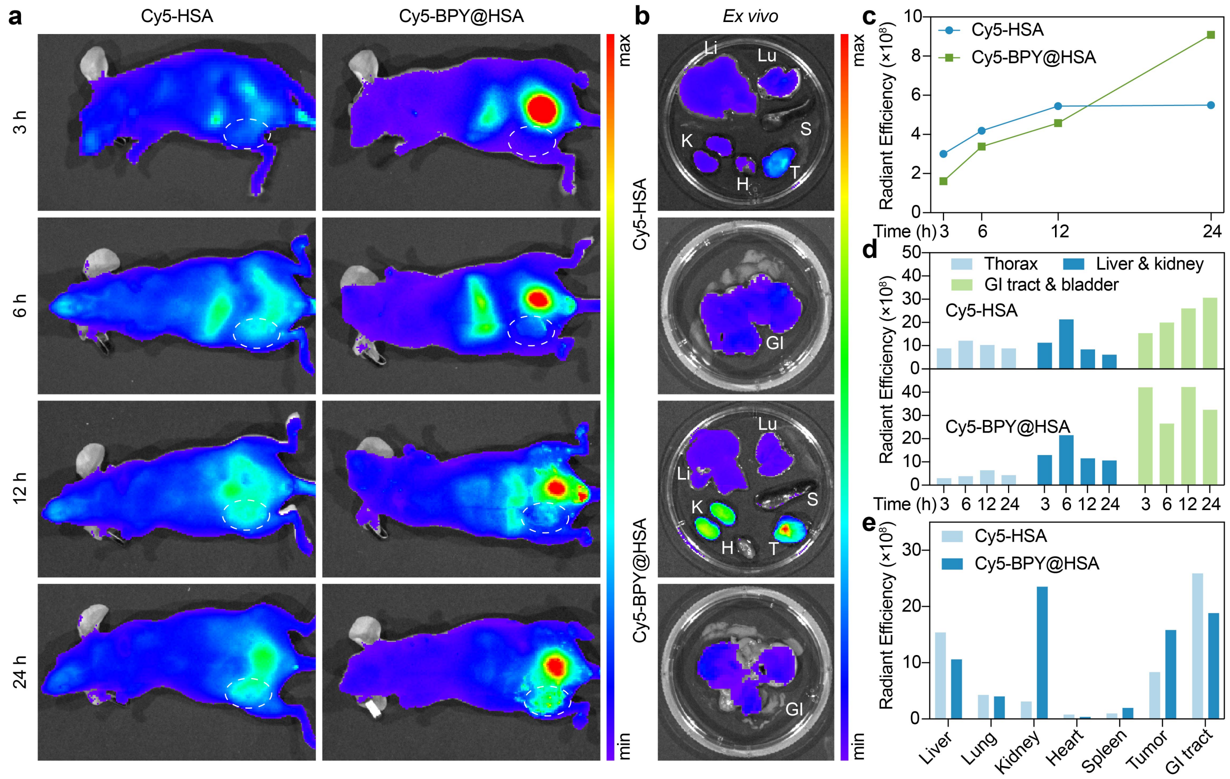

Supplementary Fig. 39. Biodistribution investigation of the Cy5-HSA and Cy5-BPY@HSA formulations on 4T1 orthotopic tumor models. a *In vivo* imaging at indicated time points post intravenous injection of Cy5-HSA and Cy5-BPY@HSA (white cycles indicate tumor areas). **b** *ex vivo* imaging of harvested major organs and tumors at 24 h post intravenous injection of Cy5-HSA and Cy5-BPY@HSA. Radiant efficacy of **c** tumor areas, **d** selected areas against time, and **e** *ex vivo* organs and tissues (n = 1).

Supplementary Note 5: Discussion for Supplementary Fig. 39

For 4T1 orthotopic tumor model, the mice were lied under anesthesia to clearly observe the tumor. As shown in Supplementary Fig. 39, Cy5-HSA formulation also presented systemic distribution (the liver was not similar with the side placed mouse in Supplementary Fig. 29, because the height and position difference would influence the acquisition focus of the camera in the instrument) while Cy5-BPY@HSA formulation exhibited preferred distribution in liver, kidney, GI tract and bladder, indicating the liver was the major organ mediating the metabolism of the Cy5-BPY@HSA and GI tract and kidney were the major routes mediating the excretion of the Cy5-BPY@HSA. For the

tumor accumulation efficacy, both formulations showed fluorescence signals on tumor sites, and Cy5-BPY@HSA presented enhanced tumor accumulation than that of Cy5-HSA.

Considering the results in Supplementary Fig. 29, the tumor accumulation of Cy5-BPY@HSA was found to be superior to that of Cy5-HSA on these two tumor models. Furthermore, moderate tumor accumulation of Cy5-HSA was observed on the 4T1 orthotopic tumor model, as opposed to the MCF-7 xenografted tumor model. It was inferred that the differences in Cy5-HSA formulation between the two models resulted from the distinct natural characteristics of each tumor model, which would be discussed in Supplementary Note 6. This result aligned with the findings revealed by the above cellular uptake mechanism experiment results. Additionally, Cy5-BPY@HSA showed a distinct fate compared to natural HSA upon entry into the bloodstream, as it targeted tumor tissue and was metabolized by the liver before being eliminated through the kidney (*via* urine) and GI tract (through bile acid or intestinal secretion).

Supplementary Note 6: Discussion for the accumulation variation of Cy5-HSA (or BPY-HSA) on different tumor models

Cy5-BPY@HSA presented excellent tumor accumulation on two tumor models, and the Cy5-HSA (BPY-HSA) showed moderate tumor accumulation on 4T1 orthotopic tumor model but MCF-7 xenografted tumor model not. The major reason that caused the difference of the therapeutic effect was the inert characterizations of different kinds of tumors, which varied in molecular, cellular, and tissue levels.

On the molecular level, it was evidenced that MCF-7 cell line does not possess caveolae-mediated pathway owing to the lack of caveolins and cavins-related molecular machinery[4], which was an important pathway for cellular uptake and it also mediated transcellular transport from blood vessels to deep tissues. In addition, the cellular uptake to Cy5-HSA (or BPY-HSA) on the levels (4T1 could uptake more) and mechanisms (4T1 depended on CLIC and caveolin, while MCF-7 only depended on CLIC) were different between MCF-7 and 4T1 cell lines (discussed in Supplementary Note 2), which caused the uptake difference. Furthermore, the infiltration ability to

deep tumor tissues was investigated by 3D tumor spheroids as well. Cy5-HSA showed better infiltration ability in 4T1 models than that of MCF-7 (Fig. 3f,k, Supplementary Fig. 19), which was resulted by the molecular machinery differences and the cellular uptake levels between the two cell lines; that was, the transcytosis ability would influence the infiltration of the formulations, however, only adequate cellular uptake of surface cells on 3D tumor spheroids to the formulation could ensure the deep infiltration. Not only a series of tumor inert factors affecting the uptake and transcytosis of the formulations in *in vitro* tumor uptake and infiltration, but the results of *in vivo* tumor accumulation also demonstrated the differential tumor target ability of the Cy5-HSA (or BPY-HSA) on the different tumor models. Sufficient tumor accumulation *in vivo* was the base of good photothermal therapeutic effect. Therefore, the BPY-HSA could exert better photothermal effect on 4T1 orthotopic tumor model than that of MCF-7. Even though the BPY-HSA presented moderate tumor accumulation on 4T1 orthotopic tumor model, the photothermal therapeutic effect was still inferior to the BPY@HSA formulations because the BPY@HSA presented much more higher tumor accumulation *in vitro* and *in vivo*.

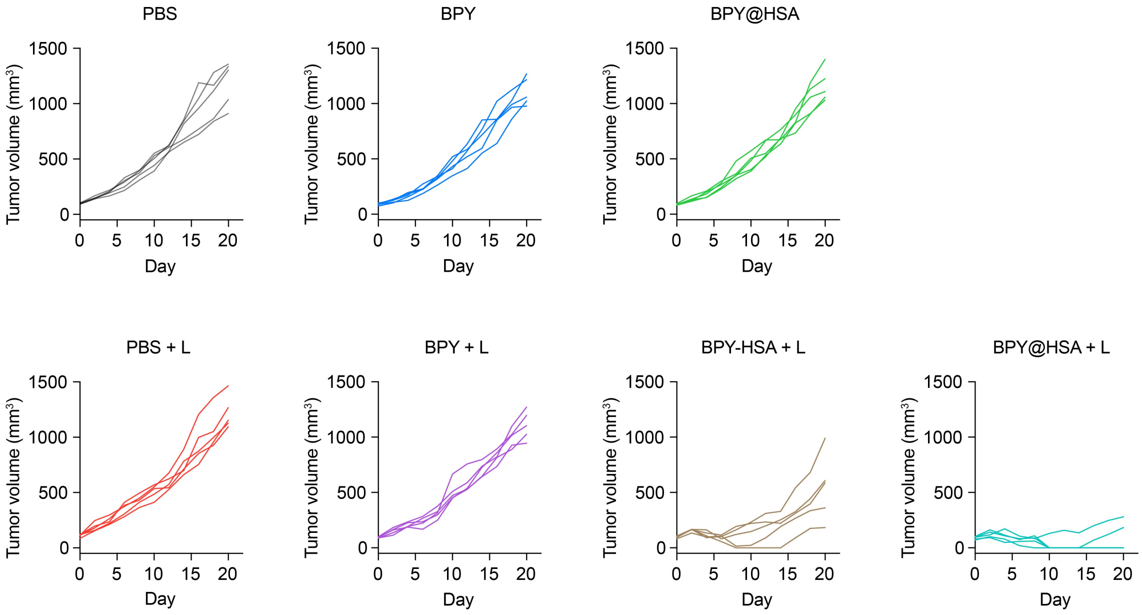

Supplementary Fig. 40. Tumor growth curves of 4T1 tumor model (*n* = 5) with different treatments indicated.

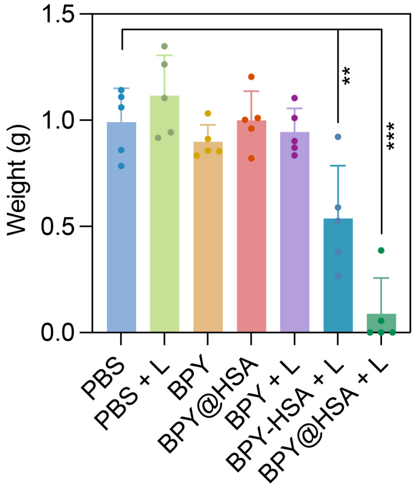

Supplementary Fig. 41. Harvested tumor weight of 4T1 tumor model (n = 5) with different treatments indicated.

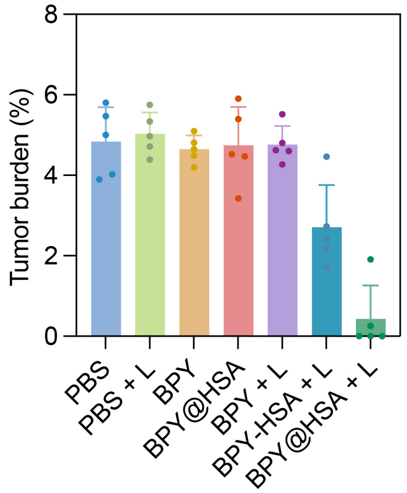

Supplementary Fig. 42. Tumor burden at the end of treatment on 4T1 tumor model (n = 5) with different treatments indicated.

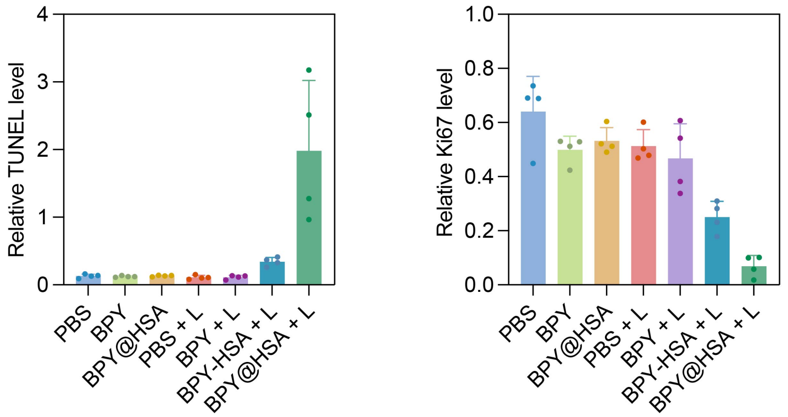

Supplementary Fig. 43. MFI analysis to TUNEL and Ki67 tumor slides of 4T1 model with different treatments indicated (n = 4).

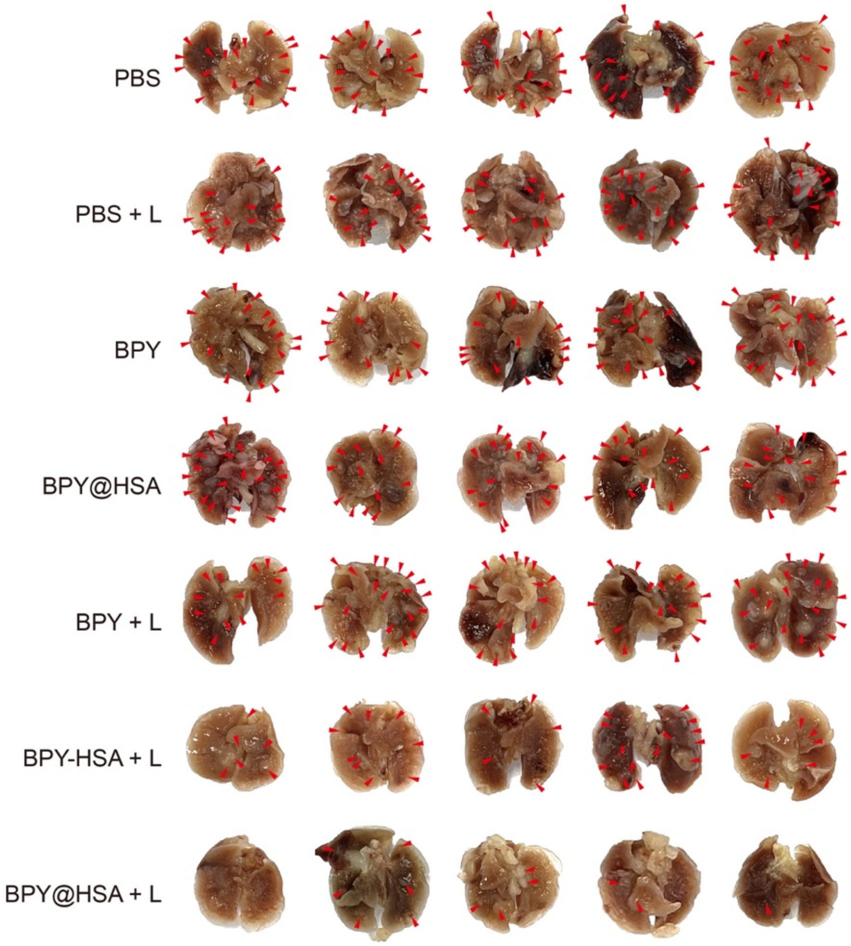

Supplementary Fig. 44. Harvested lungs for analyzing lung metastatic nodules with different treatments indicated (n = 5).

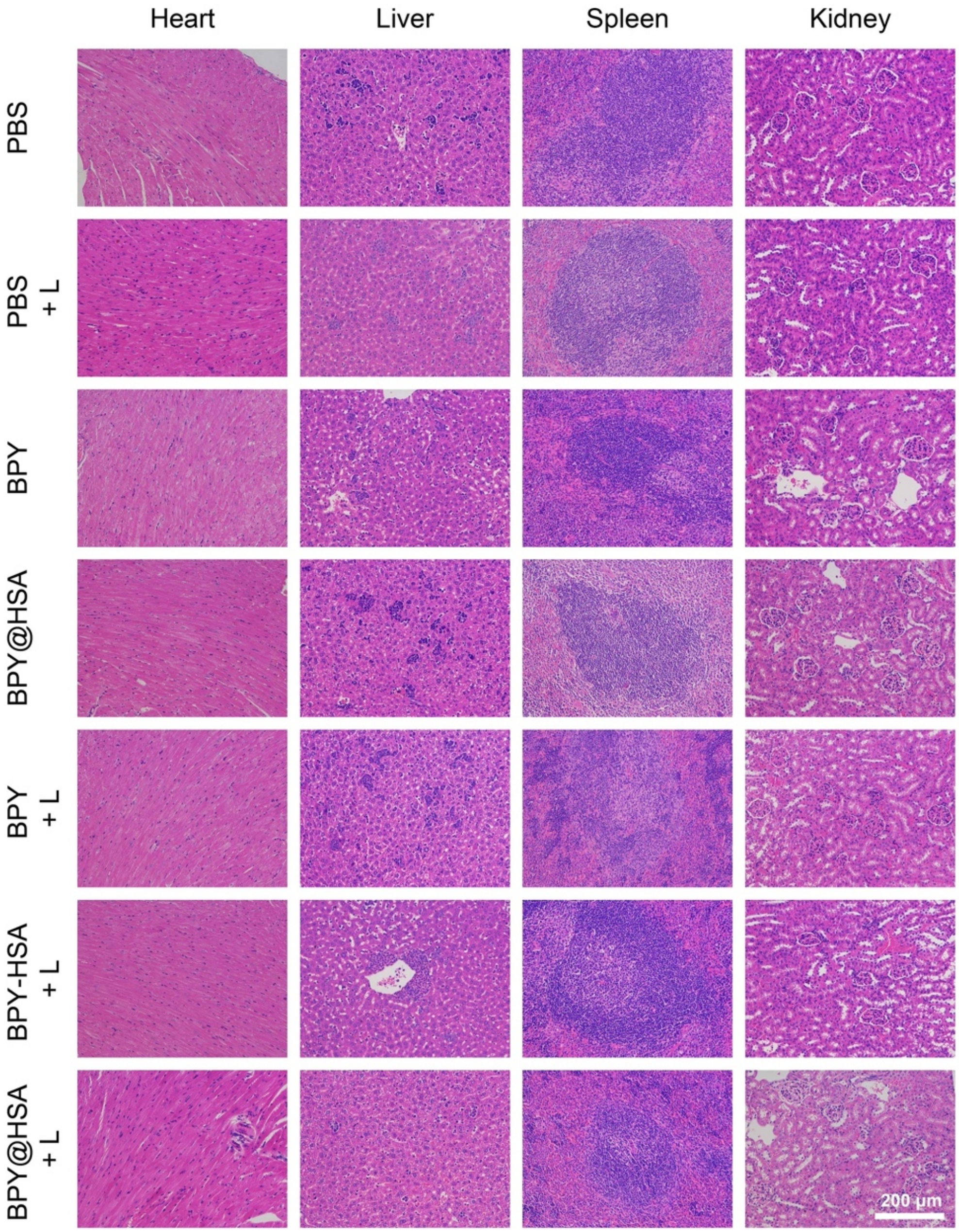

Supplementary Fig. 45. Representative H&E staining slides of major organs harvested from 4T1 model with different treatments indicated. No obvious histological damages were founded in each treatment group.

Section S3: Supplementary Tables

Supplementary Table 1. Loading capacity (LC) of several representative HSA-based platforms.

Type	Interaction	Drug	Platform	LC
Covalent binding [a]	-COOH, $-NH_2$	Ce6	HSA-Ce6[5]	1.28%
		Oxa(IV)	HSA-Oxa(IV)[5]	1.82%
		CySCOOH	HSA-CySCOOH[6]	4.1%
	-SH	monomethyl auristatin E	HSA-MMAE[7]	2%
Noncovalent binding [b]	Hydrophobic interaction	ICG	ICG@HSA-AZO[8]	3.73%
		PTX	HSA-PTX-RGD[9]	6.3%
HSA-assembled nanoparticles [c]	Programmed assemble	ICG	HSA-ICG NPs[10]	11.0%
	Self-assemble	dc-IR825, gambogic acid	HSA/IR825/GA NPs[11]	1.1%, 0.2%
HSA-based polymers [d]	Modified residues on denatured HSA	DOX	denatured HSA chain-based polymer[12]	3.91%
HSA crosslinked nanoparticles [e]	Genipin crosslinked	PTX	Genipin crosslinked HSA fragments[13]	7.0-8.3%
	Glutaraldehyde crosslinked	Sunitinib analogue	GA crosslinked HSA NP[14]	4.0%
	Cysteine crosslinked	PTX	Cysteine crosslinked albumin NP[15]	18.3%

[a] Covalent binding formulated HSA-platforms are based on covalent bonds between drugs and HSA, thus covalent binding strategy requires the drugs to have reactive groups with sulfhydryl groups or amino groups that are naturally existed on HSA protein, and the drugs containing sulfhydryl groups, maleimide groups, and NHS esters could be loaded to HSA by covalent binding.

[b] Noncovalent binding formulating strategies are based on hydrophobic interaction, electrostatic attraction, aromatic stack, and some weak bonds, which is not requiring the drugs to own covalent

binding groups. Such formulating strategies could be employed to load hydrophobic small molecular drugs, charged molecular drugs, and some biomolecules.

[c] HSA-based self-assembled nanoparticles are prepared by noncovalent interactions of HSA and drug units, which own hydrophilic parts and hydrophobic tails that could provide larger hydrophobic space for efficient drug loading.

[d] HSA-based polymers are kinds of HSA polypeptide chain derivates, which are prepared by modifying residues on denatured HSA polypeptide chains. Such polymers enlarged the molecular binding sites for loading more drugs, however, the carrier skeleton would gain the weight as well.

[e] HSA crosslinked nanoparticles require crosslink agents to serve as “glues” to bind the drugs and HSA proteins to form nanoparticles, which relate both covalent and noncovalent interactions among the agents, drugs, and HSA proteins.

Supplementary Table 2. IC_{50} values calculated from CCK-8 experiments of each treatment.

Cell line	Formulation	Laser (-)	Laser (+)
MCF-7	BPY-HSA	> 500 μg/mL	9.39 μg/mL
	BPY@HSA	> 500 μg/mL	8.37 μg/mL
4T1	BPY-HSA	> 500 μg/mL	9.81 μg/mL
	BPY@HSA	> 500 μg/mL	8.93 μg/mL
B16	BPY-HSA	> 500 μg/mL	9.04 μg/mL
	BPY@HSA	> 500 μg/mL	8.76 μg/mL

References

1. Zeng, W., *et al.* Renal-clearable ultrasmall polypyrrole nanoparticles with size-regulated property for second near-infrared light-mediated photothermal therapy. *Adv. Funct. Mater.* **31**, 2008362 (2021).
2. Jokic, T., *et al.* Highly photostable near-infrared fluorescent pH indicators and sensors based on BF2-chelated tetraarylazadipyrromethene dyes. *Anal. Chem.* **84**, 6723-6730 (2012).
3. Rennick, J.J., Johnston, A.P.R. & Parton, R.G. Key principles and methods for studying the endocytosis of biological and nanoparticle therapeutics. *Nat. Nanotechnol.* **16**, 266-276 (2021).
4. Gambin, Y., *et al.* Single-molecule analysis reveals self assembly and nanoscale segregation of two distinct cavin subcomplexes on caveolae. *eLife* **3**, e01434 (2013).
5. Yang, G., *et al.* A hypoxia-responsive albumin-based nanosystem for deep tumor penetration and excellent therapeutic efficacy. *Adv. Mater.* **31**, 1901513 (2019).
6. Rong, P., *et al.* Protein-based photothermal theranostics for imaging-guided cancer therapy. *Nanoscale* **7**, 16330-16336 (2015).
7. Liu, X., *et al.* Controlled loading of albumin-drug conjugates ex vivo for enhanced drug delivery and antitumor efficacy. *J. Control. Release* **328**, 1-12 (2020).
8. He, P., *et al.* Dual-stage irradiation of size-switchable albumin nanocluster for cascaded tumor enhanced penetration and photothermal therapy. *ACS Nano* **16**, 13919-13932 (2022).
9. Chen, Q., *et al.* Drug-induced self-assembly of modified albumins as nano-theranostics for tumor-targeted combination therapy. *ACS Nano* **9**, 5223-5233 (2015).
10. Sheng, Z., *et al.* Smart human serum albumin-indocyanine green nanoparticles generated by programmed assembly for dual-modal imaging-guided cancer synergistic phototherapy. *ACS Nano* **8**, 12310-12322 (2014).
11. Gao, G., *et al.* Molecular targeting-mediated mild-temperature photothermal therapy with a smart albumin-based nanodrug. *Small* **15**, 1900501 (2019).

12. Wu, Y., Shih, E.K., Ramanathan, A., Vasudevan, S. & Weil, T. Nano-sized albumin-copolymer micelles for efficient doxorubicin delivery. *Biointerphases* **7**, 5 (2012).

13. Ge, L., *et al.* Human albumin fragments nanoparticles as PTX carrier for improved anti-cancer efficacy. *Front Pharmacol.* **9**, 582 (2018).

14. Altintas, I., *et al.* Nanobody-albumin nanoparticles (NANAPs) for the delivery of a multikinase inhibitor 17864 to EGFR overexpressing tumor cells. *J. Control. Release* **165**, 110-118 (2013).

15. Jiang, L., *et al.* A nontoxic disulfide bond reducing method for lipophilic drug-loaded albumin nanoparticle preparation: formation dynamics, influencing factors and formation mechanisms investigation. *Int. J. Pharm.* **443**, 80-86 (2013).
