# Supplementary material for: NIR-dye bridged human serum albumin reassemblies for effective photothermal therapy of tumor
Source: Nat Commun. 2023 Oct 17;14:6567. doi: 10.1038/s41467-023-42399-9 (PMC10582160; doi:10.1038/s41467-023-42399-9)
Supplement: Supplementary file 3 — Reporting Summary [file 41467_2023_42399_MOESM3_ESM.pdf]

nature portfolio

Corresponding author(s): Lin Mei, Yanli Zhao, Hongzhong Chen

Last updated by author(s): Sep 19, 2023

Reporting Summary

Nature Portfolio wishes to improve the reproducibility of the work that we publish. This form provides structure for consistency and transparency in reporting. For further information on Nature Portfolio policies, see our Editorial Policies and the Editorial Policy Checklist.

Statistics

For all statistical analyses, confirm that the following items are present in the figure legend, table legend, main text, or Methods section.

n/a	Confirmed	
☐	☒	The exact sample size (n) for each experimental group/condition, given as a discrete number and unit of measurement
☐	☒	A statement on whether measurements were taken from distinct samples or whether the same sample was measured repeatedly
☐	☒	The statistical test(s) used AND whether they are one- or two-sided<br>*Only common tests should be described solely by name; describe more complex techniques in the Methods section.*
☒	☐	A description of all covariates tested
☒	☐	A description of any assumptions or corrections, such as tests of normality and adjustment for multiple comparisons
☐	☒	A full description of the statistical parameters including central tendency (e.g. means) or other basic estimates (e.g. regression coefficient) AND variation (e.g. standard deviation) or associated estimates of uncertainty (e.g. confidence intervals)
☐	☒	For null hypothesis testing, the test statistic (e.g. F, t, r) with confidence intervals, effect sizes, degrees of freedom and P value noted<br>*Give P values as exact values whenever suitable.*
☒	☐	For Bayesian analysis, information on the choice of priors and Markov chain Monte Carlo settings
☒	☐	For hierarchical and complex designs, identification of the appropriate level for tests and full reporting of outcomes
☐	☒	Estimates of effect sizes (e.g. Cohen's d, Pearson's r), indicating how they were calculated

Our web collection on statistics for biologists contains articles on many of the points above.

Software and code

Policy information about availability of computer code

Data collection	Particle solutions (Version 3.6.0.6376) was used to collect the zeta potential and DLS data;<br>CytExpert (Version 2.3) was used to record FCM data;<br>ZEN (Version 3.0) was used to take CLSM images;<br>viewMSOT (Version 3.8) was used to obtain PAI data;<br>Living Image (Version 4.2) was used to take Cy5 fluorescence images.
Data analysis	All the quantitative data were expressed as mean ± s.d., and the statistics were analyzed with Origin Pro 2021 software.<br>GraphPad (Version 9.4.1), FlowJo (Version 10.8.1), Origin Pro 2021, Fluke SmartView (Version 4.3), ImageJ (Version 1.54c), and Adobe Illustrator 2022 were used for data analysis and figure production.

For manuscripts utilizing custom algorithms or software that are central to the research but not yet described in published literature, software must be made available to editors and reviewers. We strongly encourage code deposition in a community repository (e.g. GitHub). See the Nature Portfolio guidelines for submitting code & software for further information.

Data

Policy information about availability of data

All manuscripts must include a data availability statement. This statement should provide the following information, where applicable:

- Accession codes, unique identifiers, or web links for publicly available datasets
- A description of any restrictions on data availability
- For clinical datasets or third party data, please ensure that the statement adheres to our policy

The source data underlying Figure 2, Figure 3, Figure 4, Figure 5, Figure 6, and Supplementary Figures are provided with this paper. The main data that support the findings of this study are available within the article, source data and its Supplementary Information. Other relevant data during the study are available for research purposes from the corresponding authors upon reasonable request.

Research involving human participants, their data, or biological material

Policy information about studies with human participants or human data. See also policy information about sex, gender (identity/presentation), and sexual orientation and race, ethnicity and racism.

Reporting on sex and gender	N/A
Reporting on race, ethnicity, or other socially relevant groupings	N/A
Population characteristics	N/A
Recruitment	N/A
Ethics oversight	N/A

Note that full information on the approval of the study protocol must also be provided in the manuscript.

Field-specific reporting

Please select the one below that is the best fit for your research. If you are not sure, read the appropriate sections before making your selection.

☒ Life sciences ☐ Behavioural & social sciences ☐ Ecological, evolutionary & environmental sciences

For a reference copy of the document with all sections, see nature.com/documents/nr-reporting-summary-flat.pdf

Life sciences study design

All studies must disclose on these points even when the disclosure is negative.

Sample size	For property measurement experiments, samples were prepared individually in triplicate unless otherwise mentioned. For in vitro studies, each group contains three individual biological replicates for evaluating the statistical significance unless otherwise mentioned. For FCM study, at least 10000 single cells were gated for analysis. For in vivo tumor treatment studies, each group contains 5 biological replicates for evaluating the tumor treatment efficacy. For other biological analysis, the biological replicates were given by n.
Data exclusions	No data was excluded from the analysis.
Replication	For property measurement experiments, samples were replicated and tested independently at least twice, and the biological replicates (n) were indicated in the figure caption.
Randomization	Randomization was used to divide up the animals for in vivo treatment study.
Blinding	No blinding was employed as the researcher performing the treatment was also responsible for the analysis.

Reporting for specific materials, systems and methods

We require information from authors about some types of materials, experimental systems and methods used in many studies. Here, indicate whether each material, system or method listed is relevant to your study. If you are not sure if a list item applies to your research, read the appropriate section before selecting a response.

Materials & experimental systems

n/a	Involved in the study
☐	☒ Antibodies
☐	☒ Eukaryotic cell lines
☒	☐ Palaeontology and archaeology
☐	☒ Animals and other organisms
☒	☐ Clinical data
☒	☐ Dual use research of concern
☒	☐ Plants

Methods

n/a	Involved in the study
☒	☐ ChIP-seq
☐	☒ Flow cytometry
☒	☐ MRI-based neuroimaging

Antibodies

Antibodies used	Anti-Ki67 Rabbit pAb (Servicebio, GB111499, dilution 1:500)<br>Cy3-conjugated Goat Anti-Rabbit IgG (Servicebio, GB21303, dilution 1:300)
Validation	The antibodies listed above are standard reagents used in the field and validated in the literature as cited on the manufacturers websites, as well as by the manufacturers data sheets themselves.<br>1. Anti-Ki67 Rabbit pAb: https://www.servicebio.cn/goodsdetail?id=3931<br>2. Cy3-conjugated Goat Anti-Rabbit IgG: https://www.servicebio.cn/goodsdetail?id=253&specificationId=204

Eukaryotic cell lines

Policy information about cell lines and Sex and Gender in Research

Cell line source(s)	4T1 and MCF-7 cell lines were purchased from ATCC (CRL-2539, HTB-22); B16 cell line was purchased from Procell (CL-0029).
Authentication	Cell lines were not authenticated.
Mycoplasma contamination	Cell lines were not tested for mycoplasma contamination.
Commonly misidentified lines (See ICLAC register)	No commonly misidentified cell lines are used in this study.

Animals and other research organisms

Policy information about studies involving animals; ARRIVE guidelines recommended for reporting animal research, and Sex and Gender in Research

Laboratory animals	Female Balb/c nude mice (6 weeks old, SPF) were purchased from Hunan SJA (Changsha, China) and raised in Laboratory Animal Research Center, South China University of Technology. Animals were housed in groups of 5 mice per IVC cage, maintained at a temperature of ~25 °C in a 40%-70% humidity-controlled environment with a 12 h light/dark cycle.
Wild animals	The study did not involve wild animals.
Reporting on sex	No reporting on sex were involved in this study.
Field-collected samples	No field collected samples were involved in this study.
Ethics oversight	All animals were handled in accordance with the policies and guidelines of the Animal Ethics Committee of Laboratory Animal Research Center, South China University of Technology (Animal Ethics Committee approval number: 2021061)

Note that full information on the approval of the study protocol must also be provided in the manuscript.

Flow Cytometry

Plots

Confirm that:

☒ The axis labels state the marker and fluorochrome used (e.g. CD4-FITC).

☒ The axis scales are clearly visible. Include numbers along axes only for bottom left plot of group (a 'group' is an analysis of identical markers).

☒ All plots are contour plots with outliers or pseudocolor plots.

☒ A numerical value for number of cells or percentage (with statistics) is provided.

Methodology

Sample preparation	For cellular uptake studies, the cells were washed with PBS and trypsinized for flow cytometry analysis. For apoptosis analysis, the cells were trypsinized for Annexin V-FITC and PI staining according to the protocol of kit manufacture, and the stained cells were dispersed in PBS for flow cytometry assays.
Instrument	CytoFLEX S
Software	Flow cytometry data were analyzed using FlowJo (v10.8.1) for macOS.
Cell population abundance	The purity of post-sort fractions is regularly measured by the software. The fractions were around 70-90%.
Gating strategy	A forward-scatter/side-scatter gate was used to gate on single cells to exclude debris.

☒ Tick this box to confirm that a figure exemplifying the gating strategy is provided in the Supplementary Information.
